# Rice Seed Germination Underwater: Morpho-Physiological Responses and the Bases of Differential Expression of Alcoholic Fermentation Enzymes

**DOI:** 10.3389/fpls.2017.01857

**Published:** 2017-10-26

**Authors:** Berta Miro, Toshisangba Longkumer, Frederickson D. Entila, Ajay Kohli, Abdelbagi M. Ismail

**Affiliations:** Genetics and Biotechnology Division, International Rice Research Institute, Makati, Philippines

**Keywords:** anaerobic germination, coleoptile, alcohol dehydrogenase, aldehyde dehydrogenase, acetaldehyde, anaerobiosis, splice-variants, post-translational modifications

## Abstract

The water-, energy-, and labor-intensive system of transplanted puddled rice (*Oryza sativa*) is steadily being replaced by direct seeding due to the progressive scarcity of these resources. However, the alternate dry direct seeding leads to competition with weeds and poor establishment when soils are flooded. Direct seeded rice capable of anaerobic germination (germination in flooded soil, AG) is ideal, which under rainfed ecosystems would also overcome waterlogging during germination. AG tolerance is associated with faster germination and faster elongation of coleoptiles, with the activities of alcoholic fermentation enzymes replacing aerobic respiration as a source of energy. To better understand the variability in the morpho-physiological responses and in the nature of the alcoholic fermentation enzymes during AG, 21 rice genotypes were studied. The genotypes Khao Hlan On (KHO) and IR42 were used as the tolerant and susceptible checks, respectively. KHO exhibited faster germination, with 82.5% of the coleoptiles emerging out of 10 cm of water within 8 days, whereas IR42 exhibited 20% germination and limited coleoptile growth. Among the test genotypes, four performed well, including two that are drought tolerant. Increased content and activity of the alcoholic fermentation enzymes, alcohol dehydrogenase (ADH1) and acetaldehyde dehydrogenase (ALDH2a and ALDH2b), was noted in KHO under anaerobic than under aerobic conditions and also in comparison with IR42 under AG. Gene transcripts for these enzymes were also more in KHO undergoing AG. However, no major differences were observed between KHO and IR42 in the critical *cis*-acting regulatory elements, such as the auxin, light, and sugar response elements, in the promoters of ADH1, ALDH2a, and ALDH2b genes. Post-transcriptional and post-translational regulatory mechanisms were implicated for the increased transcript and protein content/activity of the enzymes in KHO by observing four different transcripts of ALDH2a and a unique non-glycosylated form of ADH1 under AG. IR42 lacked the non-glycosylated ADH1 and contained only a truncated form of ALDH2a, which lacked the active site. Additionally, KHO exhibited increased activity and more isoforms for reactive oxygen species detoxifying enzymes under AG compared to IR42. These results highlight the need for a deeper functional understanding of the critical enzymes involved in AG.

## Introduction

Flooding during germination results in poor crop establishment and yield in rice and other cereal crops, as seeds of most crops are sensitive to low oxygen stress during germination and early seedling growth. Rice is the only cereal that exhibits a degree of tolerance to anaerobic conditions during germination (AG), which is limited to coleoptile emergence and partial growth, but not sufficient to overcome the stress. Flooded conditions during seed germination can be a consequence of uneven leveling of the field or early rain, or sometimes, when rice fields are purposely flooded after sowing to combat weeds.

Developing rice varieties that can germinate under flooded conditions is essential for direct-seeded system to be widely adopted by farmers. Most of the recent research has been focusing on coleoptile elongation and percent germination, which are the two most important traits known to be associated with AG tolerance. Recently, a QTL (*AG1*) was characterized and the underpinning gene, threalose-6-phosphate phosphatase (O*sTTP7*) proven to be functionally responsible for tolerance to anaerobic stress (Kretzschmar et al., 2015). The O*sTTP7* was found to be involved in sugar signaling, which in turn regulated various genes related to starch mobilization and conferred the AG tolerance trait. Other studies reported the use of GWAS to identify new regions and genes of interest for AG (Entila et al., 2014; Hsu and Tung, 2015; Zhang et al., 2017). Since most AG lines are landraces, this is a good step ahead in detecting novel genes and genetic linkages. One of the newer gene targets obtained from GWAS is *HXK6*, suggested for further studies in relation to coleoptile elongation and AG (Hsu and Tung, 2015). Another candidate gene that has been recently highlighted is a DUF domain containing protein (Zhang et al., 2017), suggested to control coleoptile length under flooded conditions. Also a recent RNAseq study of six rice genotypes highlighted genes and pathways related to coleoptile elongation and AG (Hsu and Tung, 2017). These were classified into categories of adjustments in the carbohydrate metabolism, pyrophosphate-dependent energy supply pathways, ethylene signaling pathways and cell elongation. All transcriptome, NGS and proteome analysis studies have identified enzymes for alcoholic fermentation as significantly upregulated under submerged conditions (Lasanthi-Kudahettige et al., 2007; Huang et al., 2009; Ismail et al., 2009; Narsai et al., 2009, 2015; Shingaki-Wells et al., 2011; Hsu and Tung, 2017).

Alcoholic fermentation is associated with rice seed germination under water to provide energy when oxygen is insufficient for normal respiration. This energy facilitates seed germination and coleoptile elongation to rapidly bring the shoots above the water surface to overcome anaerobic stress (Ismail et al., 2009). As the coleoptile elongates above water, processes for detoxification of the fermentation products and scavenging of the reactive oxygen species (ROS) are initiated (Ismail et al., 2009, 2012; Colmer et al., 2014; Kirk et al., 2014). Such a balanced energy generation and product detoxification is key to surviving the hypoxic or anoxic conditions through coleoptile growth.

Three main enzymes involved in alcoholic fermentation are pyruvate decarboxylase (PDC, EC 4.1.1.1.), alcohol dehydrogenase (ADH, EC 1.1.1.1.), and aldehyde dehydrogenase (ALDH, EC 1.2.1.3.). These have been previously studied in relation to rice seed germination in flooded soils (Nakazono et al., 2000; Tsuji et al., 2003; Meguro et al., 2006; Ismail et al., 2009, 2012; Estioko et al., 2014). However, regulatory mechanisms underpinning differential content and activity of these enzymes for AG are not clear.

The enzyme at the center of the alcoholic fermentation process is ADH1, which is crucial for tolerance of low oxygen stress during germination in rice (Matsumura et al., 1995, 1998; Guglielminetti et al., 2001; Shingaki-Wells et al., 2011). ADH1 rapidly reduces the toxic acetaldehyde into ethanol, which is a key step for AG. The other enzyme involved in the detoxification process is ALDH. The role of ALDH2 in rice seed germination under water has been discussed after confirmation of increase in its expression under flooded conditions (Nakazono et al., 2000). Later studies revealed the involvement of ALDH2a and ALDH2b, the two isoforms, and most studies confirmed that ALDH2a was more responsive to flooding/anoxia (Tsuji et al., 2003; Lu et al., 2005; Meguro et al., 2006; Sadiq et al., 2011). Hypotheses on the regulation of these enzymes involve decreased oxygen concentration (Nakazono et al., 2000; Ismail et al., 2009; van Dongen et al., 2009), decreased energy (Zabalza et al., 2009), cytosolic calcium (Nakazono et al., 2000; Tsuji et al., 2000), or pyruvate concentration (Kato-Noguchi, 2006; Bailey-Serres and Voesenek, 2008; Zabalza et al., 2009).

The aim of this study was to develop a better understanding of the differences in expression and functionality of ADH and ALDH during alcoholic fermentation under AG, by comparing a tolerant (KHO) and sensitive (IR42) rice genotypes. The upregulation of ADH and ALDH under AG was confirmed. Notably, however, ALDH2b isoform did not react differentially during AG in the contrasting genotypes. The upregulation of ADH1 and ALDH2a during AG in KHO compared to IR42 does not seem to depend on the respective genes’ promoters. Instead, our observations of the differential splice-variants of ALDH2a and post-translational modifications (PTMs) of ADH1 may underlie important differences in the content and activities of these enzymes. In parallel, differential activation of the ROS detoxifying enzymes in the two genotypes might contribute to the contrasting phenotype during AG. Taken together our results pave the way for exploring the avenues of splice-variants and protein modification as regulatory mechanisms underpinning differential content/activity of particular isoforms of the enzymes critical for rice seed germination under water.

## Materials and Methods

### Experimental Design

This paper has two sets of experiments, a physiological characterization with 21 genotypes and a molecular characterization with three genotypes (included within the 21 genotypes). Growth conditions of plant material for both sets followed the same protocol as detailed below. Any differences were detailed in the pertinent section.

### Plant Material and Growth Conditions

Seeds of all rice genotypes studied (Supplementary Table 1) were sourced from the International Rice Research Institute, Philippines. Seed dormancy was broken by incubating the seeds at 50°C for up to 5 days prior to sowing. Seeds (caryopses) were sown at 0.5–1.0 cm below the soil surface in the greenhouse. Separate plastic trays were used for each genotype. The soil was a clay loam mix from upland fields. Trays were watered to field capacity in aerobic treatments (non-flooded, 0 mm water level). For flooding treatment, trays were placed in submergence pools with water levels maintained at 100 mm for 10 days, or 21 days for some morpho-physiological measurements. Greenhouse yearly average conditions are: day/night cycles of 12 h/12 h; temperatures of 30°C/25°C; and humidity between 70 and 85%.

### Measurements on Seedling Morphology

Measurements were taken at 10 and 21 DAS. Percentage germination was calculated as the ratio of number of germinated seeds per genotype divided by the number of total seeds sown (*n* = 20). Germinated seeds were those with emerging radicle and coleoptile visible to the naked eye. Length measurements in cm were recorded using a ruler and in mm using a caliper (measurements detailed in Supplementary Table 2). Dry weights of shoots and roots were recorded separately from five seedlings (homogenously selected out of 10). Shoot and roots (excluding the seed) were oven-dried at 70°C for 5 days, wrapped in paper towel. Root traits were analyzed with ImageJ and WinRhizo software (Schneider et al., 2012). Roots were cut, cleaned of soil and rinsed before placing in the scanner and running a standard analysis with a volume of soil of 0.003 m^3^.

### Sample Preparation for Molecular Analyses

Seedlings grown as described above were sampled every day from day 1 until 10 DAS, immediately frozen in liquid nitrogen and stored at -80°C until use. Dry seeds were used for day 0 in both control and flooded treatments.

### DNA Extraction, PCR, and Cloning

Around 500 mg of each sample was pulverized in liquid nitrogen and DNA was extracted using the CTAB method. Kapa HiFi PCR kit was used for cloning (Kapa Biosystems). The reaction volume was 20 μL and the cycling conditions were as recommended by the manufacturer. Primer sets were: *ADH1* promoter: 5′-GATATGCTTTTCTCCACAGC-3′, 5′-GCATTCCTACTTGGACTATCA-3′; *ADH1* gene: 5′-ATCCTCTTCACCTCGCTCTG-3′, 5′-CACCTCCAGCTCCTTCTTCA-3′; *ALDH2a* promoter: 5′-TCCAATTGAAAGCGTCAAAA-3′, 5′-ACAATGCCTCTGTCCACTCC-3′; *ALDH2a* gene: 5′-ATTTCTGGTCACGCCGCAAG-3′, 5′-CGCAATCTCTGGCGTTGTTC-3′; and *ALDH2b* gene: 5′-CTCCACCTAAAATGGGAGCA-3′, 5′-GCACTCTAAACCGCTTCTGG-3′. The primers for *ALDH2a* gene were also used to check the splice variants. PCR amplicons were cloned in pCR Blunt TOPO vector (Thermo Fisher Scientific) and sent for sequencing.

### Total RNA Extraction

The method used for RNA extraction was a modification of Li and Trick (2005) method for samples with high carbohydrate content. Firstly, samples were rapidly pulverized into powder in liquid nitrogen, and immediately transferred to PureLink Plant RNA Reagent (Life Technologies) in substitution to Li and Trick’s Extraction Buffer 1. After that, the Li and Trick’s protocol was followed. A DNase treatment was also included between the isopropanol and the ethanol precipitations. The DNase used was the RQ1 RNase-Free DNase (Promega) and the treatment was performed following manufacturer instructions, with increase in the incubation time to 1–2 h at 37°C. The cDNA was synthesized immediately after the RNA was extracted (ImProm-II^TM^ Reverse Transcription System, Promega, United States).

### Quantitative RT-PCR (qRT-PCR)

A 10 μl reaction volume consisted of 1.0 μl normalized cDNA, 5 μl 2X SYBR green PCR master mix (Roche Diagnostics GmbH, Germany) and 0.4 μl of 10 mM primer for each primer pair. Reactions were run in triplicate in a 7500 Fast Real-Time PCR System (Applied Biosystems, Foster City, United States). Amplification conditions were 50°C for 2′, 95°C for 2′, 40 cycles of denaturing at 95°C for 10″ and a combined annealing and extension step at 55°C for 30″, followed by a disassociation stage from 55 to 95°C (melting curve analysis). The comparative threshold cycle (ΔΔCt) method was used to quantify the relative expression levels. Primers used were (forward and reverse): *ALDH2a* 5′TCTTCTTCAACCAGGGGCAA3′, 5′TGGCCTTCTCCACGAACTC3′, and *ALDH2b* 5′TTGAACAGGGCCCTCAGATT3′, 5′TAATCTGTCGCCACCAGTCA3′.

### Protein Extraction

About 500 mg of seeds/seedlings were ground to a powder in liquid nitrogen, and added to cold buffer composed of 100 mM TRIS pH 8.0, 2 mM DTT, and 10% glycerol. The homogenate was centrifuged at 12,000 × *g* at 4°C for 20 min. The supernatant was used as crude extract. Protein concentration was determined using the Bradford reagent (Sigma B 6916) with bovine serum albumin as standard (Bradford, 1976).

### Enzyme *In-gel* Assays

*In-gel* assays for different enzymes were conducted using the same crude extract. Thirty microgram of total protein was loaded per sample in 8% non-denaturing PAGE, and subjected to electrophoresis (20–25 mA per gel for 2 h at 4°C) prior to *in-gel* detection. All water used for buffer and solution preparations was autoclaved prior to use.

*Aldehyde dehydrogenase:* The gel was immersed in 200 mL staining solution [50 mM TRIS buffer pH 8.2, 1.5 mM NAD^+^, 0.06 mM phenazine methosulfate, 0.25 mM 3-(4,5-dimethylthiazol-2-yl)-2,5-diphenyltetrazolium bromide and 0.15 mM acetaldehyde] at 30°C for 30 min in the dark or until dark blue bands appeared.

*Ascorbate peroxidase:* Protein extracts were added with 5 mM L-ascorbate immediately after extraction and gels were run with 5 mM L-ascorbate in the running buffer. After electrophoresis, the gel was submerged in 50 mM phosphate buffer pH 7 containing 2 mM L-ascorbate for 30 min, with buffer changes every 10 min. The buffer was finally discarded and the gel was incubated in solution A (50 mM phosphate buffer pH 7, 4 mM L-ascorbate, 2 mM H_2_O_2_) for 20 min. The gel was rinsed in 50 mM phosphate buffer pH 7 for 1 min and finally incubated in solution B (50 mM phosphate buffer pH 7, 28 mM TEMED, 2.45 mM nitroblue tetrazolium until clear bands appeared in a dark purple background.

*Catalase:* The gel was washed in ddH_2_O three times for a total of 30 min, then incubated in 0.3% H_2_O_2_ for 10 min, rinsed in ddH_2_O for 2 min, and finally incubated in 2% potassium ferricyanide solution and 2% ferric chloride solution (poured at the same time on the gel, without prior mixing). After 5 min, light yellow bands appear against a dark green–blue background.

*Superoxide dismutase:* After electrophoresis the gel was incubated in the darkness for 10 min in a solution (50 mM TRIS–Cl pH 8, 13 mM EDTA, 21 mM TEMED, 0.049 mM riboflavin, 3 mM NBT), and then exposed to light until clear bands appeared against a dark purple background.

### Protein Purification

For protein purification, 500 g of fresh seedlings grown for 5 days under flooded conditions were ground to a powder in liquid nitrogen, added to cold buffer composed of 50 mM TRIS pH 8.0 and 2 mM DTT and centrifuged at 9000 × *g* for 20 min at 10°C. The supernatant was used as crude extract. The protein was isolated first with ammonium sulfate fractionation (AS, Supplementary Figure 6A). Precipitate of fractions of 25 and 75% AS were collected and resuspended in 20 mL of 50 mM TRIS pH 8.0, 5 mM NaCl, and 2 mM DTT buffer (buffer A). The resuspended fraction was loaded on a DEAE sepharose column (GE healthcare). The column was first washed with buffer A until the eluate showed no more absorbance at 280 nm and then eluted with buffer A containing linear gradient of 0.005–0.5 M NaCl (Supplementary Figure 6B). The enzyme fractions were pooled and loaded on a CM sepharose column (GE healthcare) equilibrated with 10 mM potassium phosphate buffer with 2 mM DTT (buffer B) at a flow rate of 1 ml/min. The bound protein was eluted with gradient of 0.005–0.5 M NaCl in buffer B (Supplementary Figure 6C). Fractions with activity were again pooled, concentrated by amicon filter and loaded on to Superdex 200 column (GE healthcare) equilibrated with buffer A at a flow rate of 1 ml/min for desalting (Supplementary Figure 6D). Finally, the fractions with activity were loaded into a blue sepharose 6B column (GE healthcare). The column was first washed completely with buffer A and then eluted with buffer A containing 2 mM NAD^+^ (Supplementary Figure 6E). The enzyme fractions were pooled and concentrated. The concentrated enzyme solution was stored loaded in a SDS-PAGE gel; then the bands were excised and sent for MALDI-TOF/TOF identification. After each purification step, the different fractions were tested with *in-gel* assays for acetaldehyde activity (as described above).

### Two-Dimensional Gel Electrophoresis Analysis

The lower activity band excised from the gel (∼200 μg protein) was prepared by using a ReadyPrep 2-D Cleanup Kit (Bio-Rad Laboratories, United States). The isoelectric focusing was done using PROTEAN^TM^ IEF Cell (Bio-Rad Laboratories, United States) and immobilized pH gradient (IPG) strips of 7 cm with a linear pH range from 3 to 10. Before the second dimension, the IPG strips were equilibrated in buffer. Finally, proteins were separated on 15% SDS polyacrylamide gels, followed by staining with silver stain.

### Statistical Design and Analysis

Experiments in the greenhouse were replicated three times. Treatments in all studies were arranged in a randomized split plot design with time replications. Trait differences were subjected to principal component analysis (PCA), two-way factorial ANOVA, and *post hoc* pairwise *t*-test with the Bonferroni correction. Since the samples per group were small (*n* = 5 for aerial traits and *n* = 4 for root traits), a non-parametric Kruskal–Wallis test was also performed, with equal results as with the ANOVA. Therefore, only ANOVA results are discussed in this paper. When indicated, a *t*-test was also performed, and treatment means were compared using LSD (*P* = 0.05). All statistical analyses were performed using R software (R Core Team, 2013).

### Computational Biology Analyses

Contigs were aligned using CAP3 software (Huang and Madan, 1999) and blasted using the MSU BLAST tool (Kawahara et al., 2013). Sequences were aligned with ClustalW2 software (EMBL-EBI). Protein translation sequences were obtained from the ExPASy translate tool^1^. Protein identification was performed using MASCOT software. Glycosylation sites were analyzed with NetOGlyc 4.0 (Steentoft et al., 2013).

## Results and Discussion

### Comparison of Response to AG in a Panel of Genotypes

The 21 diverse rice genotypes representing different subpopulations (*indica, japonica, aus*) evaluated for AG are listed in Supplementary Table 1. Although coleoptile length is the standard feature used in screening for AG, and QTLs/genes have been identified for this feature (Angaji et al., 2010; Septiningsih et al., 2013; Baltazar et al., 2014), there is a growing consensus that QTLs/genes affecting additional traits relevant to flooding tolerance should be identified and characterized for a likely additive effect for consequent increase in germination and survival underwater (Septiningsih et al., 2013). Thus, the relevant aerial and root morphological features listed in Supplementary Table 2 were investigated in relation to AG and growth, 21 DAS. The number of genotypes is variably referred to 20 or 21 because IR64 was not included in assessing aerial features.

A one-way analysis of variance considering the genotype and treatment as factors (Supplementary Table 3A), followed by a *post hoc* pairwise *t*-test with Bonferroni correction revealed that the only trait not significantly different among genotypes was internode number (IN; Supplementary Table 4A). Also, the features that are well known as screening approaches, i.e., number of seeds germinated (PN) and number of plants emerging above water (NEP) were expectedly significantly different among genotypes. Other features such as the mesocotyl length (ML), leaf number (LN), portion of the plant emerging above water (PAW, in cm) and plant length (PL) related to coleoptile development and plant biomass (Miro and Ismail, 2013) were also significantly different but are not known to be used as selection traits. Similar statistical treatment (ANOVA, Mean, Kruskal–Wallis results) of the root features revealed that the significantly different traits among the genotypes were root volume (ROOTVOL), surface area (SURFAREA), projected area (PROJAREA), and root length (LENGTH; Supplementary Tables 3B, 4B).

**FIGURE 1:**
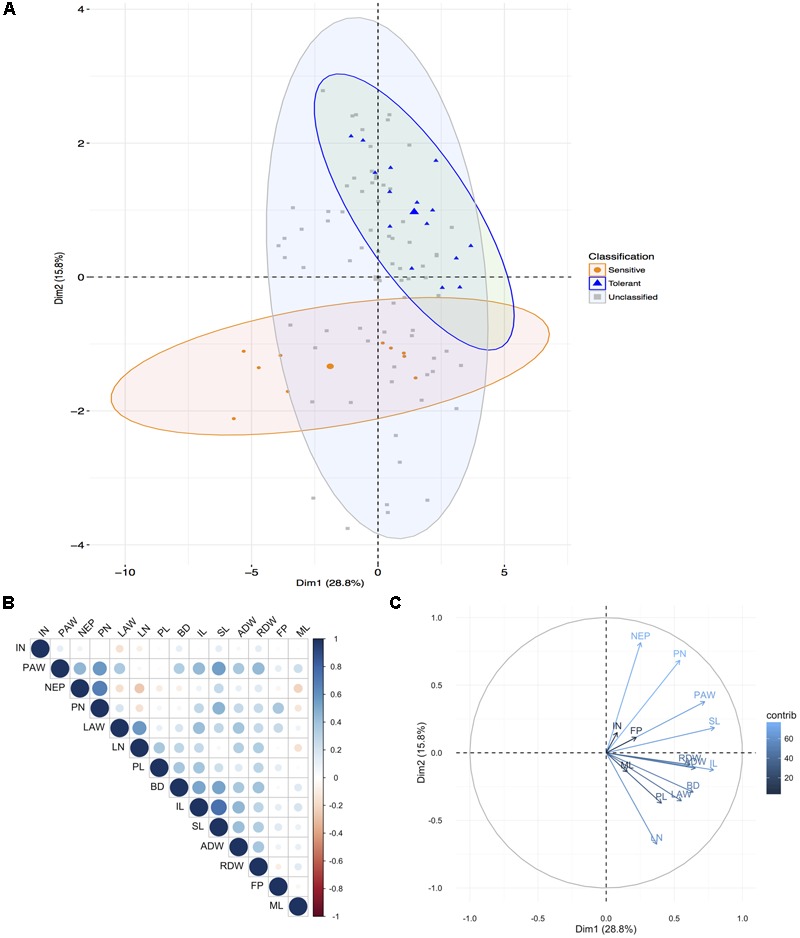
Principal component analysis for aerial traits. **(A)** Principal components (PC) of the 20 genotypes (individuals) for the 14 shoot traits (listed in Supplementary Table 1) where grouping is according to the known tolerance classification according to previous experiments: tolerant, sensitive, and unclassified (not previously been classified). Each point represents individual genotype and the three larger points represent the mean for each group. Ellipses are drawn at a 95% confidence interval. **(B)** Correlation matrix between the traits. Positive correlations are displayed in blue, and negative in red. Color intensity and circle size are proportional to the correlation coefficients. **(C)** PC plot of the 14 traits (variables) for the 20 genotypes, colored according to their percentage contribution to the component (variable cos^2∗^100/component cos^2^). Color and length of the arrow are proportional to the contribution to the component. Trait legend: IN, internode number; PAW, plants surfacing above water; NEP, number of plants emerging above water; PN, plant number; LAW, leaf portion emerging above water (cm); LN, leaf number; PL, plant length; BD, base diameter; IL, internode length; SL, shoot length; ADW, shoot dry weight; RDW, root dry weight; FP, number of floating plants; ML, mesocotyl length.

Principal component analysis of the shoot traits for the 20 genotypes under AG reiterated that the genotypes found at the extremes and thus most different were KHO and IR42 (**Figure 1A** and Supplementary Figure 2A). However, it was evident that genotypes such as ‘CO39,’ ‘Bomba,’ and ‘Khaiyan’ were positively correlated with KHO. The genotypes negatively correlated with KHO, but positively correlated with IR42 were ‘Dular’ and ‘Daw Hawm.’ Other genotypes used such as ‘Nipponbare,’ ‘Amaroo,’ and ‘Arborio’ may be classified as moderately tolerant. Interestingly, genotypes known to be moderately drought tolerant such as “Moroberekan” and “N22” fared close to “Ma Zhan Red,” and within the tolerant zone with a confidence ellipse of 95%. This relationship could be studied further to develop multi-stress tolerant varieties.

All genotypes had the innate genetic ability to respond to AG as seen when values for the measured features under normal and AG conditions were subtracted. However, it was apparent that some genotypes were better responders than others. For example, KHO, ‘Bomba,’ ‘Senia,’ and ‘CO39’ were stronger responders (**Figure 1** and Supplementary Figure 1). The remaining genotypes were relatively equally responsive among themselves but much less than the four genotypes mentioned above. Only IR42 and ‘Daw Hawm’ had a negative response to the stress and critical features such as shoot length (SL), PL, and plant number (PN), which generally increased under stress in other genotypes actually were suppressed (from increase) in these two genotypes. This can be seen from the PCA (**Figure 1** and Supplementary Figure 1), which places these two genotypes on the opposite side of the tolerant genotypes.

**FIGURE 2:**
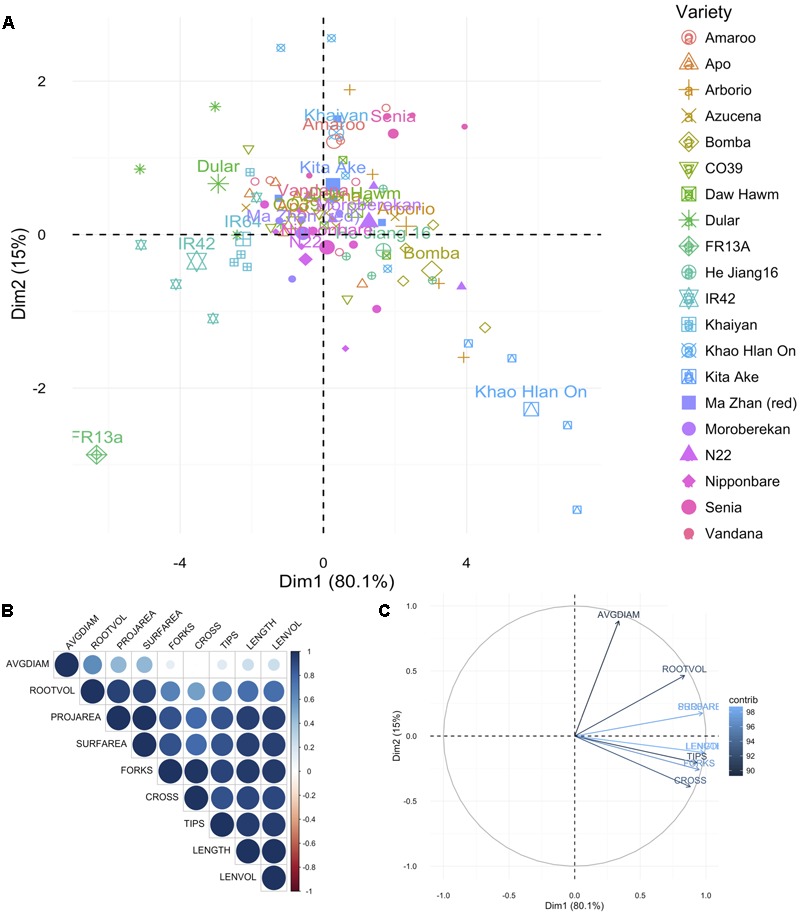
Principal component analysis for root traits. **(A)** Principal components (PC) of the 20 genotypes (individuals; listed in Supplementary Table 1) for the nine root traits, where each point represents individual genotype (four replications per genotype) and the 20 larger points represent the mean for each genotype. **(B)** Correlation matrix between the traits. Positive correlations are displayed in blue, and negative in red. Color intensity and circle size are proportional to the correlation coefficients as detailed in the legend. **(C)** PC plot of the nine root traits (variables) for the 20 genotypes, colored according to their percentage contribution to the component (variable cos^2∗^100/component cos^2^). Color and length of the arrow are proportional to the contribution to the component. Trait legend: AVGDIAM, average root diameter; ROOTVOL, root volume; PROJAREA, root projected area; SURFAREA, root surface area; FORKS, number of forks; CROSS, number of crosses; TIPS, number of tips; LENGTH, root length; LENVOL, ratio of root length to volume.

For root traits also the PCA was represented as the difference in traits measured under AG and control conditions (AG data minus control data by variety and trait). In this analysis also KHO and IR42 were found on opposite sides of the first PC dimension. Again ‘Bomba’ was positively correlated to KHO while ‘FR13A’ and ‘Dular’ to IR42 (**Figure 2A** and Supplementary Figure 2B). For the two moderately drought tolerant genotypes “Moroberekan” and “N22,” unlike the aerial traits, the root traits were not similar to the tolerant genotypes.

The most differential traits under stress for aerial parts were the SL, IL, PAW, and ADW. For roots, the most differential traits under stress were LENGTH, LENVOL, PROJAREA, and SURFAREA (**Figures 1C, 2C**). These analyses indicated that the first component in the PCA classified the genotypes according to their degree of tolerance; sensitive on the left and tolerant on the right (**Figures 1C, 2C**). Most traits, especially in roots, were positively correlated (**Figures 1B, 2B**). Only the second component separated the studied traits.

These studies suggested that along with the established methods of screening for tolerance to flooding through assessing coleoptile length, additional shoot, and root features correlated with tolerance could be included to identify genotypes that might exhibit additive effects. Taken together the aerial and root trait analysis suggested that genotypes such as Bomba (*japonica*), Senia (*japonica*), and CO39 (*indica*) may have breeding value toward discovering new QTL/genes/mechanisms for AG. These genotypes could be used in generating populations for identifying positively contributing factors for AG.

### Trait Analysis in Response to AG in Three Selected Genotypes

The tolerant KHO and the sensitive IR42, known to be contrasting for seed germination underwater, were further studied along with the moderately tolerant genotype Nipponbare (NB). Under control conditions only 10 of the 12 traits were measured since two traits namely, number of leaves above water and leaf portion above water, were specific to flooded conditions. One of the 10 features, i.e., the ML was zero in all three genotypes. For each of the remaining nine features, at least two of the three genotypes exhibited similar values (**Table 1**). Prophyllum length and root dry weight values were similar between KHO and IR42. Number of internodes and root length values were closer between NB and IR42. Seedling base diameter was the lowest in KHO, but not significantly different from the other two genotypes. For the rest of the features, values in KHO were similar to those in NB. Thus, under control conditions, values for morphological features relevant to seed germination and growth did not convincingly discriminate the tolerant from the sensitive genotype.

**Table 1:** Morphological parameters measured in ‘Nipponbare,’ ‘IR42,’ and ‘Khao Hlan On’ at 21 days under control conditions.

Control	Nipponbare		IR42		Khao Hlan On	
	Germination 75%		Germination 90%		Germination 95%	
	Average *n* = 5	*SD*	Average *n* = 5	*SD*	Average *n* = 5	*SD*
Total no. leaves	3	0	**2.2**	0.4	3	0
Mesocotyl length	0	0	0	0	0	0
Seedling base diameter	1.45	0.13	1.42	0.25	**1.27**	0.24
Length first internode	9.48	1.1	**6.95**	0.57	11.27	0.95
Number of internodes	1	0.63	0.8	0.4	0.6	0.49
Prophyllum length	**17.27**	0.87	9.13	3.72	9.47	1.68
Total shoot length	24.82	1.37	19.8	1.53	29.07	3.56
Root length	11.96	0.8	**9.46**	0.86	14.44	1.59
Aerial parts dry weight	23.82	1.95	**13.36**	3.17	23.7	3.67
Root parts dry weight	**9.24**	1.68	5.3	1.02	6.6	1.7

*Bold numbers show significant differences between varieties from the *t*-test at *P* = 0.005.*

Under AG conditions IR42 exhibited least values for 10 of the 12 features (**Table 2**). The number of internodes in IR42, i.e., one, was identical to KHO and NB. Only the IR42 mesocotyl was significantly longer than that of KHO. Most importantly, the germination percentage for KHO remained similar under the two conditions while it dropped significantly for IR42, with a germination rate drop from 90% to 20%, of which only 10% emerged above the water level. NB germination rates also dropped, but not as drastically as in IR42. These results clearly separated the tolerant KHO and the sensitive IR42 and reiterated their contrasting responses to AG (Ismail et al., 2009; Estioko et al., 2014).

**Table 2:** Morphological parameters measured in ‘Nipponbare,’ ‘IR42,’ and ‘Khao Hlan On’ at 21 days under flooded conditions.

Submergence	Nipponbare		IR42		Khao Hlan On	
	Germination 42.5%		Germination 20%		Germination 82.5%	
	Emergence 42.5%		Emergence 10%		Emergence 80.5%	
	Average *n* = 5	*SD*	Average *n* = 5	*SD*	Average *n* = 5	*SD*
No. leaves above water	1.8	0.4	1.5	0.5	**2.8**	0.4
Leaf portion above water	**17.6**	4.84	**9.38**	4.8	**25.4**	2.99
Total no. leaves	2.8	0.4	**2.25**	0.43	2.8	0.4
Mesocotyl length	2.47	0.63	**2.98**	0.87	2.6	0.08
Seedling base diameter	1.66	0.17	**1.19**	0.22	1.61	0.19
Length first internode	**12.74**	2.81	**9.5**	1.64	**17.56**	1.16
Number of internodes	1	0	1	0	1	0
Prophyllum length	22.43	2.02	**9.27**	1.87	25.91	2.47
Total shoot length	**29.32**	6.7	**22.1**	4.36	**38.74**	2.41
Root length	10.54	1.4	**3.5**	0.97	15.42	4.18
Aerial parts dry weight	22.58	6.45	**11.84**	5.68	30.84	5.38
Root parts dry weight	3.92	1.37	2.75	1.67	**5.9**	1.9

*Bold numbers show significant differences between genotypes from the *t*-test at *P* = 0.005.*

When values of the seedlings’ morphological features under control and AG were compared, it emerged that KHO had higher values than NB and IR42 in all, but the ML. Result of this analysis also indicated that all features were positively affected in KHO except root dry mass, though the decline was insignificant. Although some features were positively affected in NB and IR42 as well, the positive effect was not as strong as in KHO. However, NB and IR42 also exhibited decrease in total number of leaves, aerial parts dry weight, root length, and ‘root dry weight under AG conditions.

Under control conditions, values for five morphological features ‘seedling base diameter,’ ‘prophyllum length,’ ‘aerial parts dry weight,’ ‘total number of leaves,’ and ‘root dry weight’ in KHO were less than or equal to IR42 and/or NB. However, under AG, values for these features were higher in KHO than those for IR42 or NB. Maximum ‘fold’ increase in KHO under AG conditions was noted in ‘prophyllum length,’ followed by ‘first internode length,’ ‘total shoot length,’ and ‘aerial parts dry weight.’ Thus, for these features the genotype × treatment effect was highly pronounced in KHO. These traits are most likely contributing to tolerance of AG conditions in KHO. The prophyllum length and biomass are both advantageous for rice seeds to germinate and grow under flooding (Miro and Ismail, 2013). Consequently, ‘first internode length,’ ‘total shoot length,’ ‘aerial parts dry weight,’ and ‘prophyllum length’ are useful features to discriminate between AG tolerant and sensitive genotypes; however, % germination under water, and emergence above water are still better in terms of classification and reproducibility.

### Molecular Analysis of the Two Critical Enzymes

**FIGURE 3:**
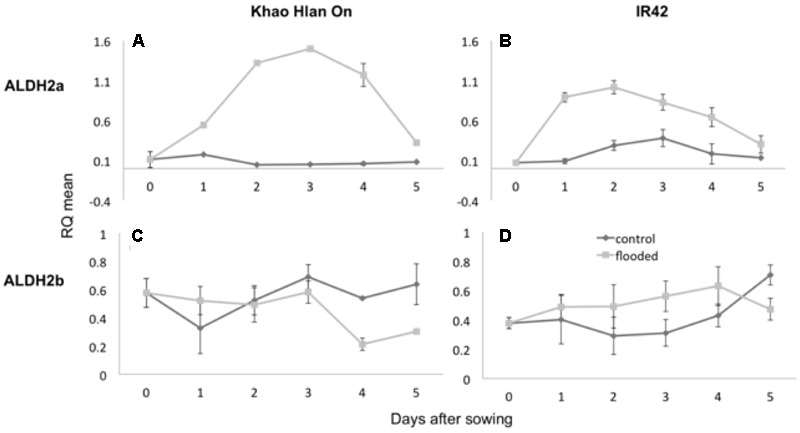
Variation in expression of *ALDH2a* and *ALDH2b* genes in ‘Khao Hlan On’ and ‘IR42’ genotypes, in both control and flooded conditions. **(A)** qRT-PCR results for *ALDH2a* in ‘Khao Hlan On’; **(B)** qRT-PCR results for *ALDH2a* in ‘IR42’; **(C)** qRT-PCR results for *ALDH2b* in ‘Khao Hlan On’; **(D)** qRT-PCR results for *ALDH2b* in ‘IR42.’ Light gray coloring represents submerged conditions and dark gray represents control conditions. SEM error bars are displayed for each time point, with three replications per time point. Relative mRNA level was normalized to the corresponding ubiquitin mRNA expression.

Aldehyde dehydrogenase and alcohol dehydrogenase enzyme activities are critically associated with AG (Guglielminetti et al., 2001; Ismail et al., 2009; Shingaki-Wells et al., 2011). Transcript content for *ALDH2a* and *ALDH2b* was ascertained by qRT-PCR in the contrasting genotypes KHO and IR42 under the control and AG conditions. In KHO *ALDH2a* expression increased 1.6-fold under AG until day 3. In IR42 it increased only up to 1.1-fold until day 2 before it started decreasing (**Figure 3**). For *ALDH2b* although there was a trend for increase in its transcript under AG in both genotypes, the increase was insignificant. These results were in accordance with earlier reports in the same two genotypes, where *ALDH2a* quickly increases under AG in KHO (Estioko et al., 2014).

Despite a minor trend for increase of *ALDH2a* transcript in IR42, the ALDH2a protein was not detected in IR42 as reported by Estioko et al. (2014). In the same study, ALDH2b protein content was similar in the two genotypes under the aerobic or anaerobic conditions. Similar results were found for *ALDH2a* and *2b* gene expression by Tsuji et al. (2003). Increased transcript and protein content of ALDH2a in the tolerant genotype under AG conditions suggested it to be the functional and critical isoform.

Transcript analysis by qRT-PCR, and cloning and sequencing revealed that under flooded conditions, KHO had three different splice forms of *ALDH2a*, whereas IR42 had only one (Supplementary Figure 3). The single transcript from IR42 did not contain the active site of the protein, which could explain the lack of immuno-detection signal in the study by Estioko et al. (2014). This transcript had an alternative start site at position 421 (in relation to the CDS of the splice form *LOC_Os02g49720.2*) and could be a target of the ‘nonsense-mediated decay’ (NMD) process of transcript degradation, which is a regulatory mechanism for transcripts that need not be translated (Houseley and Tollervey, 2009). The truncated splice form was not observed in control conditions. The longest splice form of *LOC_Os02g49720.1* was not found in either KHO or IR42 under both AG and control conditions.

**FIGURE 4:**
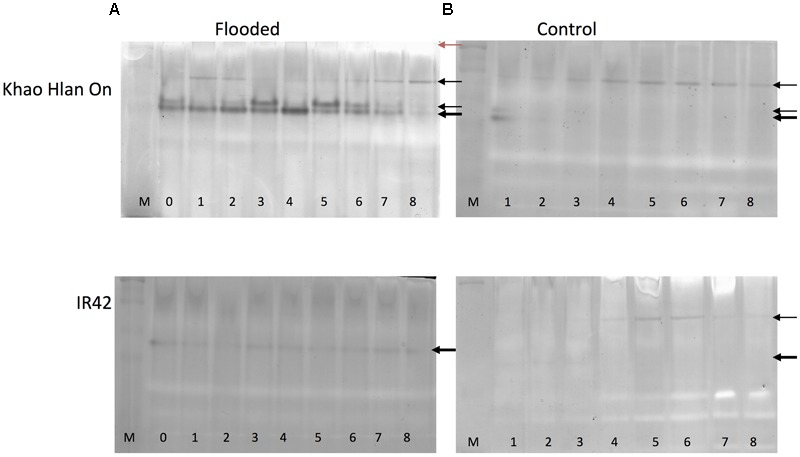
*In-gel* assay for acetaldehyde metabolism for ‘Khao Hlan On’ and ‘IR42’ in flooded and control conditions. **(A)** Dry seeds were directly sown in soil under 100 mm water (flooded) or **(B)** in soil watered to field capacity (control). Samples are loaded from 0 to 8 days in flooded and from 1 to 8 days in control, M is a dual color protein marker. In tolerant genotype ‘Khao Hlan On’ there is a dominant enzyme activity in the form of a doublet that metabolizes acetaldehyde in flooded conditions (thick black arrow). This same enzyme is also present in ‘IR42’; however, the activity is weaker and only one band is present. Another enzyme activity is visible at a higher molecular weight in both ‘Khao Hlan On’ and ‘IR42’ in both flooded and aerobic conditions. Enzyme activity that appears as white bands (red arrow) is discussed in the text.

In order to assess the correspondence between the *ALDH2a* transcript and protein content, *in-gel* activity assay for acetaldehyde metabolism was used. This analysis resulted in substantial differences in KHO and IR42 in terms of the presence/absence of the dark activity bands under the control and AG conditions (**Figure 4**). Most prominent differences were, firstly, that under AG conditions, KHO exhibited a doublet of the ca. 60 kDa band (represented by the solid white arrowheads in **Figure 4**), which was visible only as a single band in IR42. Secondly, KHO exhibited a ca. 95 kDa band under control and AG conditions (white arrowhead with black outline), which was somewhat pronounced under AG. However, IR42 completely lacked this band under AG conditions whereas it appeared faintly under control conditions only 4 days after germination. Thirdly, the AG-specific doublet in KHO clearly exhibited a sequential increase till 5 DAS followed by its decrease. Along with the dark bands, there were white bands as well (indicated by solid black arrowheads in **Figure 4**). Once again, these were more pronounced in KHO under AG. In IR42, these became pronounced again only 5 days after germination, in control conditions.

Immuno-blot assay with monoclonal anti-ALDH2 antibody was used to assess which bands were really the ALDH2. Although the expected ca. 95 kDa band was readily identified, the high molecular weight white band of more than ca. 250 kDa also hybridized, while the AG-specific ca. 60 kDa doublet yielded no signal in KHO or IR42 (Supplementary Figure 4). These results identified one set of enzyme activity bands to be due to ALDH2. Immuno-detection-based protein bands for ALDH2 were earlier identified with similar pattern under control and AG conditions (Tsuji et al., 2000; Estioko et al., 2014). However, our results could overlay the activity bands on the Western blot signals and correctly identify the activity bands due to ALDH2. The high molecular weight white bands caused by ALDH2, as revealed by Western blot signal, suggest an oligomeric form of the enzyme ALDH2a.

An attempt at *in situ* activity detection for ALDH colored the tips of the expanding coleoptile and the expanding root, more so in the tolerant KHO, indicating that the enzymes were active in the rapidly growing tissues of the plant (Supplementary Figure 5). This could be explained as an indication to facilitate the alternative energy supply to the metabolically very active tissues growing under AG.

### The AG-Specific Bands Are Due to an Alcohol Dehydrogenase

To better understand the nature of the AG-specific doublet, enrichment of the doublet in the native protein extract was standardized from the 4 DAS KHO seedlings. A four-step sequential process, using protein precipitation and chromatographic techniques led to the purification of the doublet (Supplementary Figure 6) as shown in the activity assay gel (Supplementary Figure 6E, lane 7). This doublet was excised from the gel and characterized through mass spectrometry. Both bands were identified to be the same alcohol dehydrogenase (ADH1) with a mass of 40,958 Da, calculated pI of 6.20, protein score of 71, expect of 0.00035 and 10 peptide matches covering 79% of the protein sequence. The molecular mass data, together with the corresponding putative peptide details that lead to the identification of the protein as ADH1, are described in Supplementary Table 5 and the MASCOT identification peak in Supplementary Figure 7.

Transcript expression analysis of the *ADH1* gene in KHO indicated that it had higher expression under AG conditions in comparison to that in IR42 (Ismail et al., 2009). In aerobic conditions, expression was similar between the two genotypes (data not shown). These results were in agreement with previous studies (Ismail et al., 2009; Estioko et al., 2014). Indeed the importance of ADH in AG tolerance has been elaborated earlier (Takahashi et al., 2014). In addition, the *in-gel* assay for ALDH and ADH revealed higher activity of these two enzymes in KHO. Also, *Km* for both ALDH and ADH was higher in KHO than in IR42 under flooded conditions (Estioko, 2010).

### Understanding the Doublet Band

Protein doublets mostly occur due to one of the following reasons; translation products of alternative splice forms, signal peptide processing or protein post-translation modifications. The putative ADH identified by mass spectrometry (ADH1_ORYSI, Mass: 40.958 kDa) maps to *LOC_Os11g10480* (Supplementary Figure 8), which codes for ADH1 protein. Three splice variants are predicted for this gene (*LOC_Os11g10480.1, LOC_Os11g10480.2*, and *LOC_Os11g10480.4*). The *LOC_Os11g10510* and *LOC_Os11g10520* codes for ADH2 and ADH3 protein, respectively, with the predicted splice variants of two for the first gene (*LOC_Os11g10510.1* and *LOC_Os11g10510.2*) and three for the second gene (*Os11g10520.1, Os11g10520.2*, and *Os11g10520.3*). The protein products of two splice variants in each of the two (*LOC_Os11g10480.1* and *LOC_Os11g10480.2*; *LOC_Os11g10520.1* and *LOC_Os11g10520.2*, respectively) are close to the predicted molecular weight (40.9 and 37.4 kDa; 40.7 and 37.2 kDa, respectively), which may form the protein doublet on the gel. Concordance in the original predicted molecular weight of 40.9 kDa to the LOC_Os11g10480.1 protein suggested that the bands may belong to the two splice forms of LOC_Os11g10480. However, qRT-PCR-based attempts at observing the variant CDS in KHO under AG were not successful when tested with different sets of primers. Additionally, qRT-PCR for ADH2 (*LOC_Os11g10510*) and ADH3 (*LOC_Os11g10520*) were also performed to check the possibility that the smaller band would belong to a splice form from one of these genes, but there was no amplification. All splice forms were also tested through PCR with 5′ and 3′ UTR primers. The lack of an amplification of any shorter form suggested that the doublet might not be the result of a splice variant.

The predicted protein of neither LOC_Os11g10480, LOC_Os11g10510 nor LOC_Os11g10520 contain a signal peptide, which can generally be expected due to the cytoplasmic location of ADH. Hence, signal peptide processing also cannot be the reason behind the doublet.

The option of protein PTMs causing the doublet was explored by *in silico* prediction of the possible PTMs of the ADH. Common PTMs that readily differentiate the same protein as multiple bands on the gel are protein acetylation, methylation, myristoylation, phosphorylation, glycosylation, and ubiquitination. The *in silico* analysis predicted a single *O*-glycosylation at position T120 and multiple myristoylation and phosphorylation sites of the ADH protein sequence of NB, the reference genome.

**FIGURE 5:**
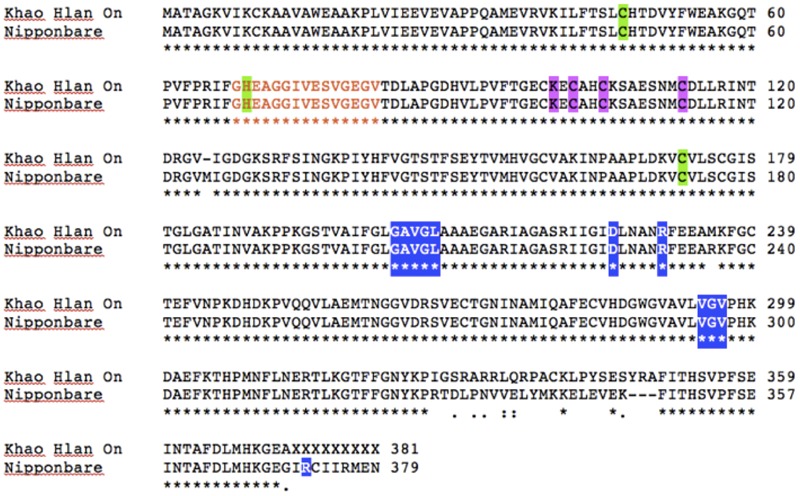
ADH1 protein sequence analysis between ‘Khao Hlan On,’ IR42 and ‘Nipponbare.’ ClustalW pairwise alignment of ‘Khao Hlan On’ protein sequence translated from the CDS cloning against “Nipponbare” sequence. Both ‘Nipponbare’ and IR42 shared the same protein sequence. Orange letters mark the active site (residues 68–82); green shading indicates the catalytic zinc binding sites (residues 47; 69; 72 KHO and 73 IR42/Nipponbare), pink shading the other zinc binding sites (residues 100, 102, 105, 113), and blue shading NAD^+^ binding sites (residues 203–207, 225, 230, 294–296 for KHO; and 204–208, 226, 231, 295–297, 372 for IR42/Nipponbare).

**FIGURE 6:**
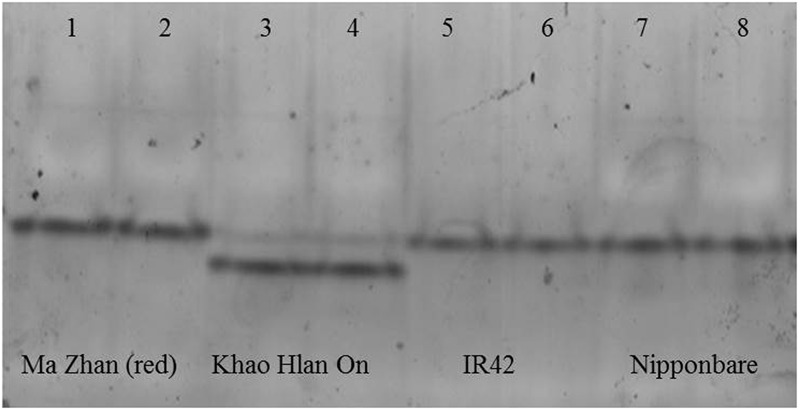
*In-gel* assay for acetaldehyde metabolism in different varieties in flooded conditions at day 6 after sowing. From left to right, tolerant genotypes are ‘Ma Zhan (red)’ (lanes 1, 2) and ‘Khao Hlan On’ (lanes 3, 4), sensitive variety IR42 (lanes 5, 6) and moderately tolerant ‘Nipponbare’ (lanes 7, 8). Each sample was loaded in two contiguous wells. All genotypes share the upper band, but the doublet is unique to Khao Hlan On within the varieties analyzed. The lower band of Khao Hlan On has stronger activity than the upper band.

**FIGURE 7:**
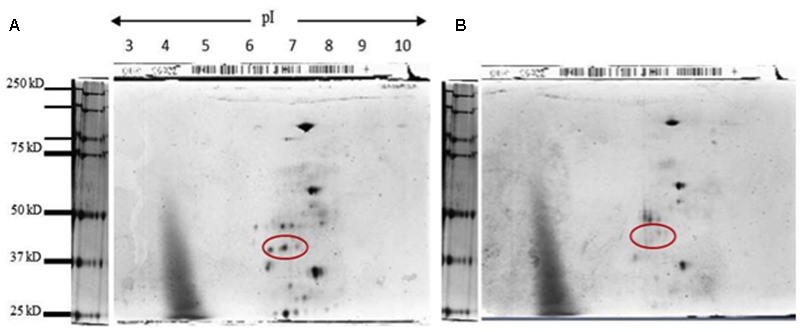
Two-dimensional denaturing gel for KHO and IR42 activity band. **(A)** Silver staining of a 2D denaturing gel for ADH activity band eluted from KHO; and **(B)** IR42. The extraction was made from seedlings grown under submerged conditions at day 4. The sample was then run in a 7 cm strip ranging from pH 3 to pH 10, and then in a denaturing gel to separate by molecular weight. To avoid sample mixing, three gels were run simultaneously in the same tank: one with the marker, one with the KHO sample and one with IR42 sample. The red circle shows a series of three spots for the putative phosphorylated moieties of the ADH1 from KHO which are absent in IR42.

To assess if such PTM could happen in KHO and/or IR42, *LOC_Os11g10480* CDS was cloned and sequenced from the two genotypes. There were four SNPs between IR42 and NB in the CDS region. Predicted protein showed that only one of these SNPs translated into a non-synonymous change of V291I. There were more non-synonymous SNPs between KHO and NB and these translated into amino acid changes shown in **Figure 5**. However, none of the changes were within the dehydrogenase domain, or the NAD^+^ or zinc binding sites. Protein modifications predicted in IR42 and NB ADH were identical. However, changes in the KHO protein sequence led to differences in the predicted PTMs such that in KHO the T120 *O*-glycosylation could not happen; there were four additional possible phosphorylation sites and one additional myristoylation site. These additional PTMs could not account for the doublet in KHO because the PTMs lead to slower movement of the protein and hence would result in the upper band of the doublet, which was anyway common to the three genotypes (**Figure 6**). This common upper band could not be due to *O*-glycosylation since KHO ADH is predicted not to be *O*-glycosylated. It could not be due only to myristoylation because it would need to be demyristoylated to lead to the lower band, and myristoylation is an irreversible process. Although AG-specific dephosphorylation could lead to the lower band in KHO, given that the ADH1 in KHO putatively has four additional phosphorylation sites, its phosphorylation status and pattern would need to be uniquely regulated in order to result in a band of molecular weight similar to IR42 and NB under control conditions. To confirm protein PTMs the ADH1 lower activity band was eluted from the gel and run on a 2D denaturing gel. The isoelectric focusing during the first dimension separated the modified proteins based on the altered charge profile. For example, a protein population differentially phosphorylated at its multiple putative phosphorylation sites would sequentially become more negatively charged. In the second dimension these modified proteins were separated according to the molecular weights. Phosphorylated proteins would appear as a series of spots along the horizontal axis around the similar molecular weight region on the vertical axis since the SDS gel will not show the small change in molecular weight (Halligan, 2009). An online tool^2^ can predict and generate a virtual 2D gel image for the input protein. When we compared the online analysis and image (Supplementary Figures 9A,B) and the ‘experimental’ 2D gel image for ADH1 from KHO (**Figure 7A**), it was easy to identify the putatively modified ADH1 spots (red circle). These were missing in IR42 (**Figure 7B**). The minor discrepancy in pI and *Mw* between the predicted and experimental spots could be due to the non-ideal experimental conditions. This data only proves the existence of PTMs but not the nature of these PTMs, which, however, is rather likely to be phosphorylation because protein modification by phosphorylation, if possible, is very common under submergence. However, they could also be acetylation (Nallamilli et al., 2014). Nevertheless, **Figure 6** shows that there is a high possibility of an AG-specific PTMs leading to the lower band of ADH1 in KHO since the intensity of the upper band decreases in comparison to NB and IR42. Due to the possibility of *O*-glycosylation in IR42 and NB but not in KHO, the most likely scenario on the common upper band is that it is a combination of the possible PTMs, including phosphorylation, perhaps identical in IR42 and NB but different in KHO, yet leading to a band of similar molecular weight. This is then most likely dephosphorylated to result in the lower band in KHO under AG. Under anoxia, dephosphorylation of sucrose synthase in maize roots has been proposed as a strong regulatory mechanism for sugar metabolism in response to stress (Subbaiah and Sachs, 2001).

Interpretation of the ADH1 doublet in terms of PTMs also rests on the polyacrylamide gel running conditions. The mobility of the modified proteins can change depending on whether the protein mass is more important, as in SDS gels, or the charge on the protein has a role, as in the native gels. The enzyme activity gels were native gels where even heavier proteins can migrate faster due to larger anionic charge. Also, structural changes in the modified proteins, especially the glycosylated ones, can make heavier, more compact proteins run faster. The *O*-glycosylation of the *O*-GlcNAc kind at a single residue may not change the mobility of the glycosylated protein on the SDS-gel (Hart and Akimoto, 2009). However, proteins with additional glycosylation may run faster on a native gel, most likely due to compaction. Therefore, it is important to note that although the glycosylation prediction tools did not predict *O*-glycosylation in the full length ADH1 in KHO, the peptide mass discrepancy in the MASCOT results when analyzed, predicted two positive sites other than the T120 of IR42 and NB (Supplementary Table 6). Hence, the possibility remains that the lower band could be an AG-specific *O*-glycosylation of KHO. An increased tendency for glycosylation under AG stress was noted for some glycolysis related glycoproteins in flooded soybean roots (Mustafa and Komatsu, 2014). Apparently *O*-GlcNAc addition is a dynamic protein modification that can affect plant response to environmental, hormonal and developmental signals (Olszewski et al., 2010). The role of *O*-glycosylation as regulator of enzyme activity, both as activator or inactivator is known (Bond and Hanover, 2015). Despite the lack of a signal peptide directing it to protein-glycosylation-organelles such as Golgi bodies, ADH glycosylation may not be a conundrum, as proteins glycosylated in the cytoplasm by cytoplasmic *O*-GlcNAc transferases are known (Hart and Akimoto, 2009) while myristoylation and phosphorylation are cytoplasmic PTMs. Protein residues targeted for phosphorylation and *O*-glycosylation, particularly *O*-GlcNAc addition, are common and one modification disallows the other at the same residue in eukaryotic proteins (Hart et al., 2007, 2011), however, both processes are dynamic and responsive to environmental signals. Only a broad and deep characterization of the AG-specific form of the ADH in KHO can dissect its PTMs and its potential role in AG tolerance in this robustly tolerant genotype. Therefore, AG-specific alterations in PTMs seem to be the reason for the appearance of the lower band of the ADH doublet in KHO. ADH is an extensively studied enzyme in varied systems but this is the first time that PTMs could be implicated in generating additional forms of the protein.

A central role has long been proposed for ADH1 under AG through its functional importance, which is reiterated by its upregulation seen under AG through the transcriptome (Lasanthi-Kudahettige et al., 2007; Narsai et al., 2009; Mohanty et al., 2012) and proteome analyses (Sadiq et al., 2011). It is further supported through the clear genetic linkage between the gene and the trait as established through studies on the rice *alcohol dehydrogenase 1*-deficient (*rad*) mutant (Takahashi et al., 2011; Edwards et al., 2012; Mohanty et al., 2016), which clearly associates impaired coleoptile growth under submergence to the lack of ATP caused by the absence of ADH1 activity. However, studies using QTL mapping (Angaji et al., 2010; Septiningsih et al., 2013; Baltazar et al., 2014) have not picked up this region/gene most likely due to low marker density around the ADH region, population structure, or due to trait selection. In this connection it is important to note that among the 3,000 sequenced rice genomes there is a single non-synonymous SNP only in 309 accessions, only 5 of which are *japonica*. Nearly one-third of the accessions have no or less than four SNPs along the promoter and the CDS and none of those SNPs are non-synonymous. Thus there is limited allelic variation in *ADH1*. This is perhaps also the reason for a below threshold peak detected through GWAS around the region of ADH1 and ADH2 genes, with weak association in the subpopulations japonica-aro panel (Entila et al., 2014). This panel was composed of temperate and tropical japonicas as well as aromatic genotypes (diversity panel 1; *n* = 158). The panel for indica-aus (diversity panel 1; *n* = 128) did not yield any peak around the ADH region on chromosome 11. Another study on AG also detected below threshold peaks along chromosome 11, however, they were not further studied (Hsu and Tung, 2015). A similar situation was found in Zhang et al. (2017) were some markers appeared as significant on chromosome 11, but they were either out of the ADH1 region, or were suboptimal. In the light of our results, future research can attempt to establish the genetic basis of an ADH1 gene deficient in protein PTMs as causative of susceptibility.

Our results suggest that one of the reasons for the lack of clear QTLs and GWAS peaks implicating ADH in AG could be the fact that there is no sufficient allelic variation in ADH. This is borne out by analysis of ADH1 in the 3K rice genome sequences^3^. The hypothesis that follows is that allelic variation in enzymes that are involved in PTMs of ADH could underlie differential response. Although this hypothesis also needs to be tested in future research, it is encouraging to note that kinases and phosphatases are known to be associated with AG (Zhang et al., 2017) and underlie some QTLs/GWAS peaks known for AG in rice (Hsu and Tung, 2015). The much discussed AG related candidate gene CBL-interacting protein kinase (CIPK25) is actually a serine/threonine protein kinase which has alcohol dehydrogenase as one of its protein–protein interacting partners at a weighted PCC of 0.519 as assessed through the rice protein interaction prediction site RiceFREND^4^. This CIPK25 is far more variable in the 3K sequences when compared to ADH1.

The last aspect is borne out by preliminary *in silico* analysis of CIPK25, which may be the kinase phosphorylating ADH1. This connects with the *in silico* as well as an *in vitro* 2D-gel experiment wherein the results are similar and they provide strong evidence for phosphorylation-mediated modification of the alcohol dehydrogenase in the tolerant variety. This result goes a long way in supporting our hypothesis that there may be a link between PTMs of alcohol fermentation enzymes and tolerance.

### Analysis of *Cis*-acting Regulatory Elements in ALDH2a and ADH1 Promoters

Differential expression of the *ALDH2a and ADH1* gene transcript and protein under control and AG conditions implicated differences in the *cis*-acting regulatory elements (CAREs) of the gene promoters. Thus the 1 kb upstream sequences of the genes for *ALDH2a* and *ADH1* were cloned and sequenced from KHO to be compared with such sequences available for NB from the reference genome sequence.

The number of CAREs clearly varied in the 1 Kb upstream sequences. There were 121 and 119 CAREs in the upstream sequence of *ADH1* in NB and KHO, respectively. Qualitatively most CAREs were shared between the two genotypes but quantitatively some motifs abounded in one or the other variety (Supplementary Figures 10A,B). Major differences were seen in the auxin, cytokinin, and nodulin related CAREs, which were present only in KHO, while the CCAAT-Heat Shock CARE was present only in NB. The CAREs uniquely present in KHO relate to root specificity/traits and might contribute to upregulating the ADH1 through better response of the promoter to these factors, which are differentially regulated under AG conditions (Rijnders et al., 1996 for auxin; Huynh et al., 2005 for cytokinin). Overall, *ADH1* regulation seemed to be related to light response in both genotypes (18% of the motifs in KHO and 17% in NB). The second most abundant group of motifs was of CAAT and CACT elements (8 and 9%, respectively, in KHO and ‘NB’). Both genotypes had elements related to response to O_2_, low CO_2_, and sugar levels. Curiously, binding sites for transcription factors (TFs) related to dehydration response were more (ca. 5%) compared to those for TFs related to other stresses (ca. 2–3%). Comparative excess of the ethylene response element (ERE) in KHO was noteworthy, especially together with the uniquely present auxin response elements. These two hormones have been identified to increase coleoptile elongation related to cell extension in rice (Breviario et al., 1992) and ethylene-response elements have been recently linked to genes responding to hypoxia (Sadeghnezhad et al., 2015).

The NB *ALDH2a* promoter sequence had a larger number of ERE/AP2, Aux, and GARE motifs than KHO (Supplementary Figures 10C,D). Also, NB had an important proportion of sugar related binding sites, in particular linked to α-amylase 3 and some NAM/NAC binding elements in NB that were not present in KHO. However, KHO had the oxygen, light or phosphate modulated TF binding sites, which were absent in NB and there were more MYC/MYB binding elements in KHO. These elements were also identified in a study where CAREs were analyzed in the ADH1 *rad* mutant (Mohanty et al., 2016). They also found that MYB and bZIP are the main CAREs found in genes altered in expression in the *rad* mutant and, therefore, related to AG through coleoptile elongation under submergence. They also reported that MYB and ERE genes are upregulated.

When CAREs were compared between *ADH1* and *ALDH2a* promoters of KHO, most were common between the two genes (Supplementary Figure 10D). Almost all common CAREs, except those related to light response, were present in higher numbers in *ALDH2a* and these included critical elements such as the MYB/MYC TF binding sites, NODULIN elements, O_2_ responsive, AuxRE, etc. The *ALDH2a* promoter uniquely possessed elements directly related to the anaerobic response, root hair, phosphate stress, etiolation, and starvation related elements while missing the sugar related elements of the *ADH1* gene. Reports suggest that *ADH1* is essential for carbohydrate metabolism in the embryo and endosperm (Takahashi et al., 2014) and related to sugar induction, however, the mechanisms are not clear (Koch et al., 2000). Additionally, five CAREs found in *ADH1* promoter of KHO, but not of NB were also found in the *ALDH2a* promoter of KHO. These were CATATGGMSAUR, NODCON2GM, OSE2ROOTNODULE, GT1GMSCAM4, and INRNTPSADB. Overall, both *ADH1* and *ALDH2a* had many more elements related to coleoptile elongation and energy generation in KHO, supporting the observed induction under AG conditions.

### Activities of ROS Detoxifying Enzymes under AG Conditions

Apart from ALDH and ADH, another set of enzymes linked to AG is the set that scavenges the ROS. The *in-gel* assays for ROS scavenging enzymes revealed that these were more active in the tolerant KHO than in the sensitive IR42 under flooded conditions (Supplementary Figure 11). These results correlated with total activities measured for the same genotypes by Ella et al. (2011). *In-gel* assays for APX and CAT showed higher number of enzymatic forms from 6 DAS onward in KHO, nearly coinciding with the coleoptile lengthening-mediated re-aeration phase (Supplementary Figure 11). The activities of APX and CAT remove the toxic H_2_O_2_ converting it to O_2_ and H_2_O mostly during the re-aeration phase. Since KHO starts elongating the coleoptiles above the soil from day 4, it is possibly 6–8 DAS when the coleoptiles reach oxygenated zones and ROS scavenging enzymes become more active to protect the seedling from post-anoxic injury from ROS formation by higher O_2_ levels, (Meguro et al., 2006). The enzymes were not present in IR42 under AG conditions. These enzymes are highly inhibited under flooded conditions (Yan et al., 1996), but that was not the case in KHO (Supplementary Figure 11). The *in-gel* assays for SOD showed that different isozymes were active in all treatments and genotypes. However, SOD activity was low in IR42 during AG. SOD activity produces H_2_O_2_ from the superoxide radical known to be generated under hypoxia and re-oxygenation (Blokhina et al., 2003). Accumulation of such oxygen radicals is damaging for the survival and viability of IR42 at a depth of 100 mm of water, especially for membrane lipids since increased SOD and CAT activities correlated with lower membrane lipid peroxidation (Yan et al., 1996; Ella et al., 2011). This, in turn, was associated with higher seedling growth and survival of the tolerant genotypes and of sensitive pre-treated genotypes. Previous research pointed at a coordinated regulation between ALDH2 and ROS enzymes, suggesting the reduction of one would favor the increase of the others (Chen et al., 2007; Xue et al., 2012). Our results confirmed that first ALDH2a was highly induced, and by the time at day 7 when its activity started to diminish, the activity of ROS scavenging enzymes CAT and APX started to increase (Supplementary Figure 11).

## Conclusion

Fast germination and coleoptile elongation are essential for rice seedling to survive flooding during germination and early seedling establishment. One of the major bottlenecks for coleoptile elongation is the metabolic reactions of alcoholic fermentation. Recent research showed a relationship between AG tolerance and a threalose-6-phosphate phosphatase (O*sTTP7*) which regulates signaling through the T6P/sucrose ratios (Kretzschmar et al., 2015). However, regulatory mechanisms and interactions that allow tolerant rice genotypes to develop a coleoptile when others do not even germinate are not fully understood. Knowledge on the genes and the mechanisms involved in this process will allow for selection of breeding lines with enhanced tolerance of these adverse conditions during germination and early seedling establishment. Both ADH1 and ALDH2 showed higher content and activity in the tolerant KHO than in the sensitive IR42. ADH1 seemed to be post-translationally regulated, potentially by an *O*-linked glycosylation whereby the protein fluctuates between the *O*-glycosylated to non-glycosylated (the predominant) form, which may underlie modulation of the enzyme activity. ALDH2a, on the other hand, seemed more regulated by splice forms, which were stress specific. In addition to that, both enzymes had ethylene and auxin response elements; the hormones intrinsically linked to coleoptile elongation. By examining the genes of interest in a pathway, we provide new avenues for queries on the mechanistic aspects of the pathway, suggesting a common activation under stress. However, these avenues should be further explored and validated. In addition, ROS scavenging enzymes were more active in KHO than in IR42. These activities relate to increased metabolism under flooded conditions and have a strong link to tolerance. Moreover, they are directly related to detoxification when the coleoptile reaches the more aerated zone at the point of emergence from the water. These findings provide mechanistic and biological evidence of the linkage between the tolerant phenotype with its genotype.

## Author Contributions

BM undertook the physiology, molecular biology, enzyme activity, and computational analyses. TL undertook the protein purification and part in the molecular biology. FE took part in the physiology and molecular biology analyses. AK, AI, BM, and TL planned the research, design of experiments and handled the interpretation and preparation of the manuscript.

## Conflict of Interest Statement

The authors declare that the research was conducted in the absence of any commercial or financial relationships that could be construed as a potential conflict of interest.

## Footnotes

1 http://web.expasy.org/translate/

2 http://proteomics.mcw.edu/promost.html

3 http://snp-seek.irri.org/

4 http://ricefrend.dna.affrc.go.jp/

## Abbreviations

ADH1: alcohol dehydrogenase 1, EC 1.1.1.1
AG: anaerobic germination (which refers to reduced oxygen during germination)
ALDH2: aldehyde dehydrogenase 2, EC 1.2.1.3
APX: ascorbate peroxidase, EC 1.11.1.11
CAT: catalase, EC 1.11.1.6
DAS: days after sowing
KHO: Khao Hlan On
NAD: nicotinamide adenine dinucleotide
SDS-PAGE: sodium dodecyl sulfate polyacrylamide gel electrophoresis
SOD: superoxide dismutase, EC 1.15.1.1
