# Supplementary material for: Rice Seed Germination Underwater: Morpho-Physiological Responses and the Bases of Differential Expression of Alcoholic Fermentation Enzymes
Source: Front Plant Sci. 2017 Oct 26;8:1857. doi: 10.3389/fpls.2017.01857 (PMC5662645; doi:10.3389/fpls.2017.01857)
Supplement: Supplementary file 1 [file Table_1.DOCX]

Supplementary Figures


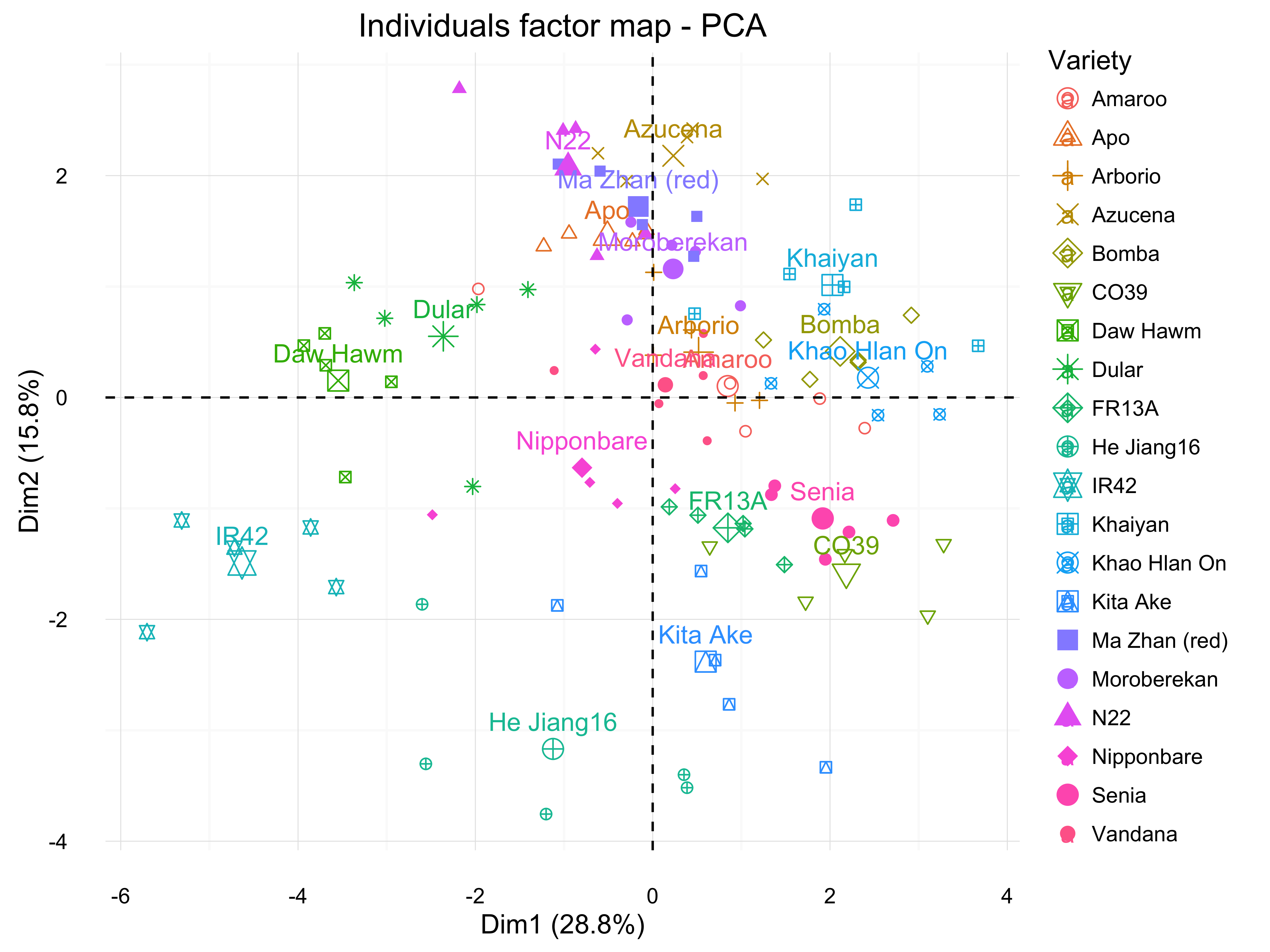


**Supplementary Figure 1.** Principal component analysis for aerial traits for the 20 varieties analyzed.


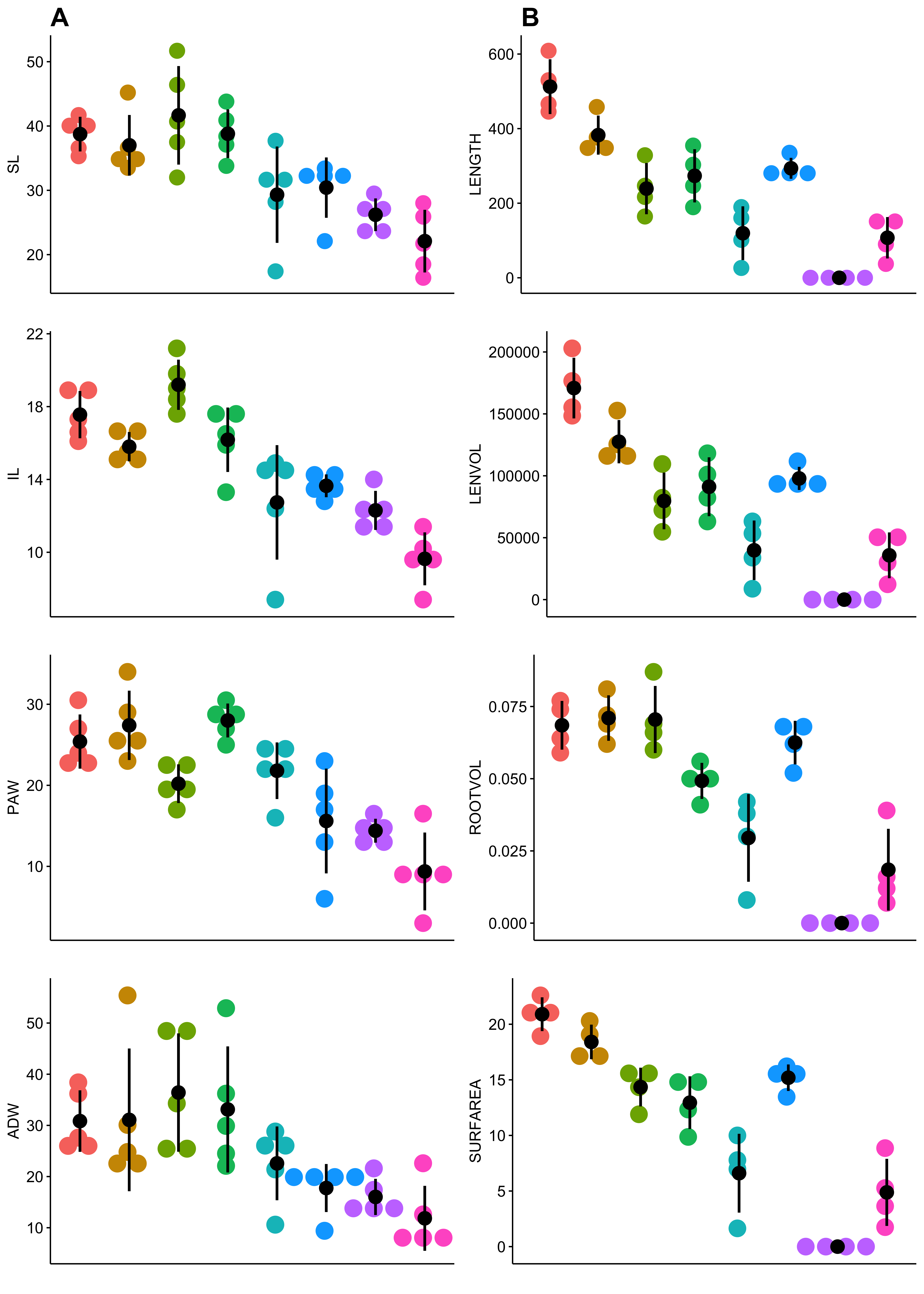


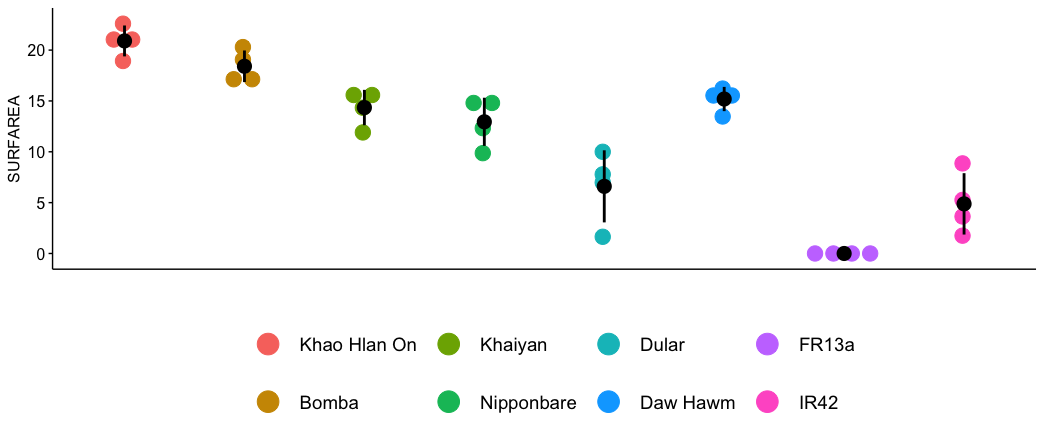


**Supplementary Figure 2.** Dotplots representing the genotypes and the traits that showed highest correlation with the first principal component dimension for aerial traits (A) and for root traits (B). Varieties are color coded according to the legend below each graph. Traits analyzed are: SL shoot length, IL internode length, PAW portion of plant emerging above water, ADW aerial dry weight, LENVOL ratio of root length into volume, LENGTH total root length, ROOTVOL root volume, SURFAREA root surface area.


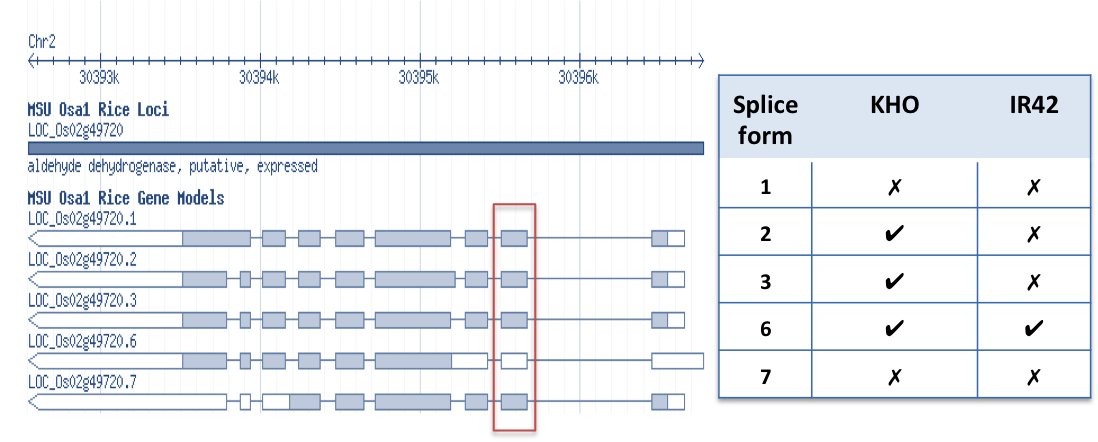


**Supplementary Figure 3. Differences in splice variants found in A*LDH2a* in KHO and IR42 in flooded conditions.** On the left, the predicted forms from the Nipponbare sequence extracted from the MSU website. On the right, table representing the splice forms identified from the cloning of RT-PCR products of KHO and IR42 in flooded conditions. The red box indicates the active site of the protein that confers DH activity.


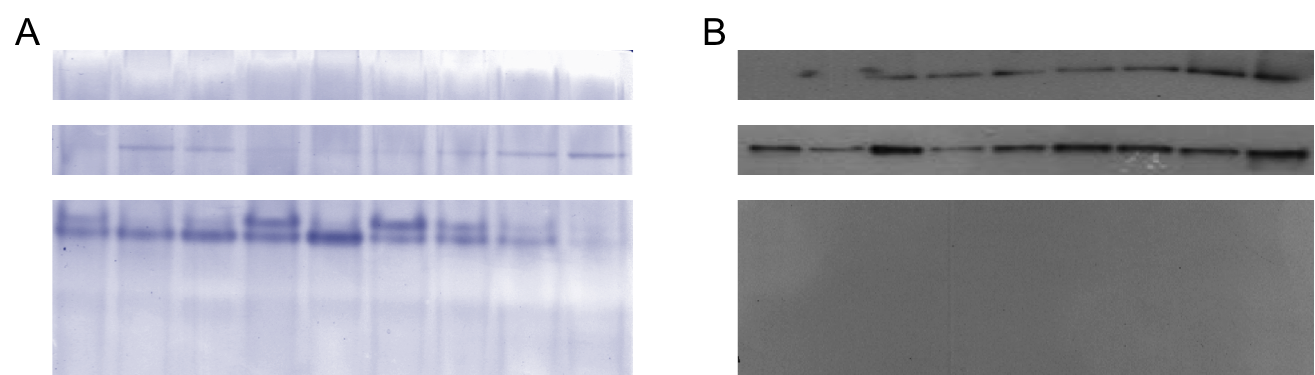


0 1 2 3 4 5 6 7 8

0 1 2 3 4 5 6 7 8

1

2

3

1

2

3

**Supplementary Figure 4. Images of (A) in gel acetaldehyde activity assay and (B) immunoblot assay raised against ALDH2 monoclonal antibody.** Lanes represent days after submergence from 0 to 8 days. The gels were loaded from the same sample and run in parallel. The cuts were realized after the enzymatic assay and before blotting, respectively at around marker positions 100kDa and 75kDa. The resulting fragments labelled in the gel were from: (1) 250kDa to >100kDa, (2) 100kDa to >75kDa and from (3) 75kDa to the end of the gel. The dark activity bands in fragments 1 and 2 matches with the immunoblot detection of the ALDH2 protein.


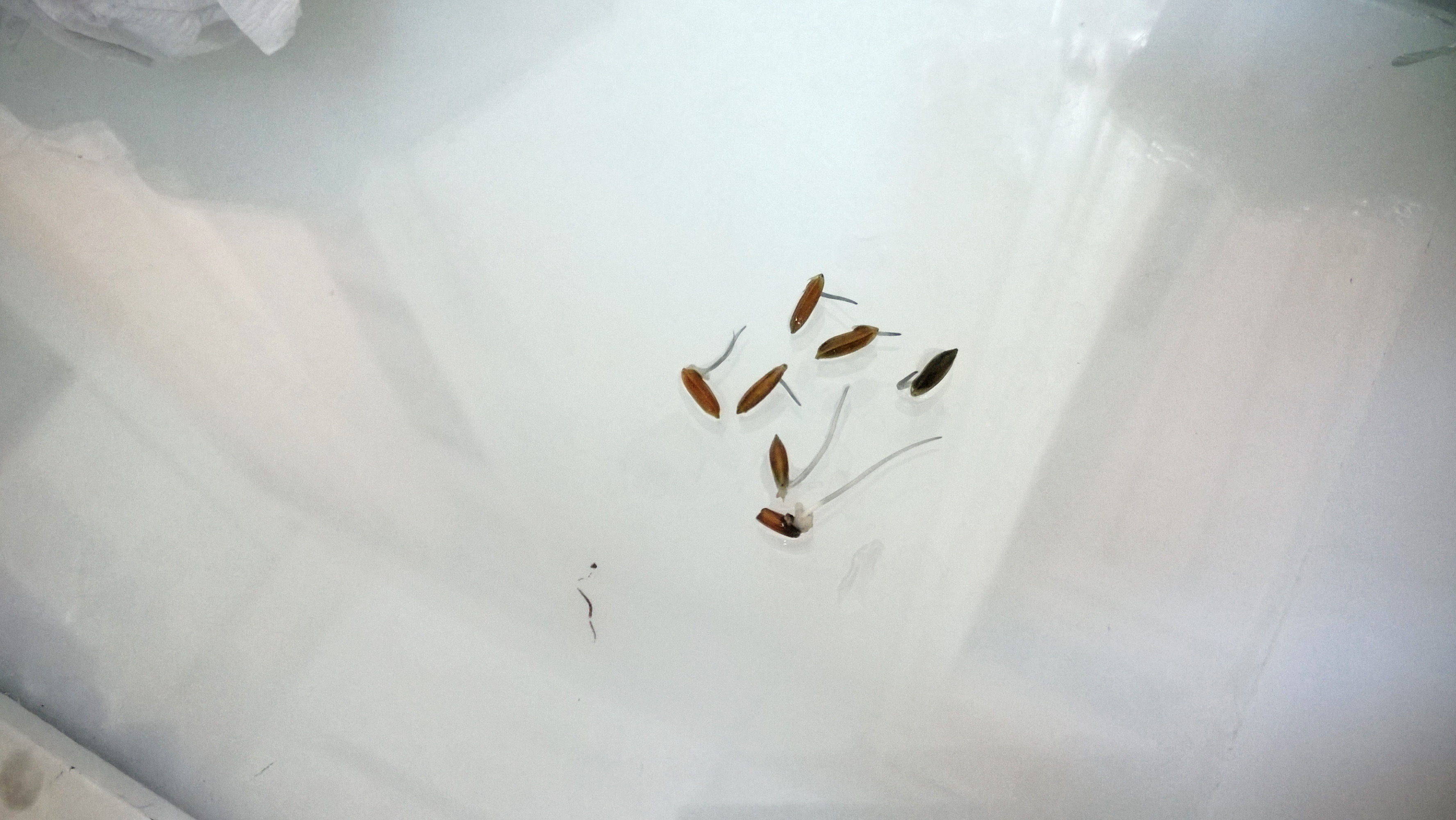


**Supplementary Figure 5. Images of in situ acetaldehyde activity assay in KHO.** The seedlings were submerged in the same reaction solution as in the in gel assays. The container was kept in the dark for about 1 minute before the coloring appeared.


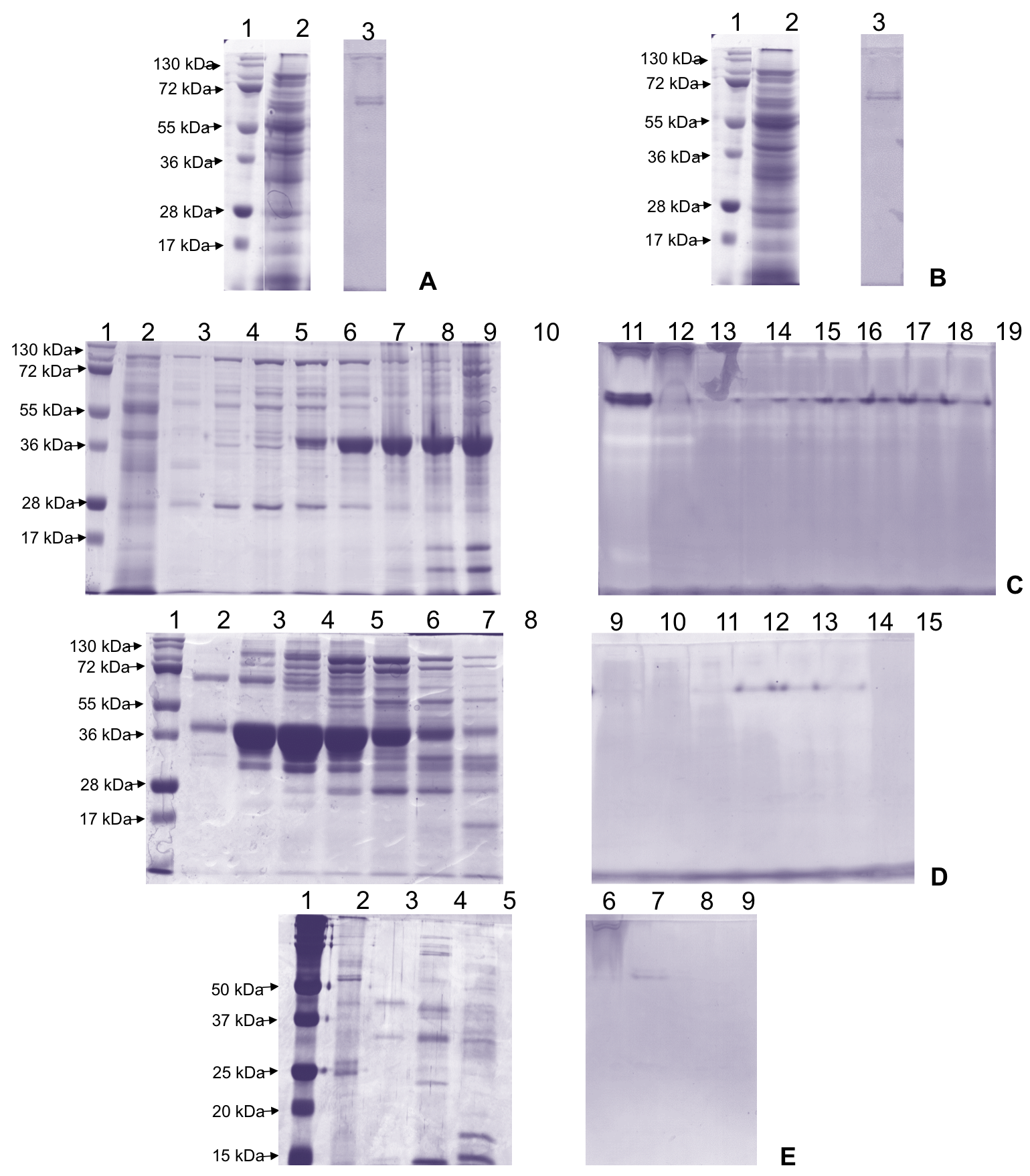


**Supplementary Figure 6. Protein purification and identification overview.** The protein was enriched sequentially using ammonium sulfate fractionation, ion- exchange chromatography, gel filtration chromatography and affinity chromatography. After each purification step, the different fractions were resolved in SDS-PAGE for comassie staining and in native PAGE for in-gel enzymatic assays. The fraction(s) showing activity were pooled together and passed to the next step. The protein activity was found in the 25-75% ammonium sulphate fraction (panel A), in the DEAE sepharose flow through (panel B), in the CM sepharose eluted fractions, and in blue sepharose eluted fractions. After the blue sepharose, the activity bands were excised from the gel and send for sequencing. Panel A: Lane 1; protein molecular weight marker (PM), 2; 25-75% ammonium sulphate fraction, and 3; corresponding zymogram. Panel B: lane 1; PM, 2; DEAE FT and, 3; corresponding zymogram. Panel C: Lane 1; PM, 2; CM sepharose FT, 3-10; eluted fractions, 11-19; corresponding zymogram. Panel D; Lane 1; PM, 2-8; Superdex S 200 eluted fractions, 9-15; corresponding zymogram. Panel E; Lane 1; PM, 2; blue sepharose FT, 3-5; eluted fractions, 6-9; corresponding zymogram.

Supplementary Figure 7. Intensity versus mass-to-charge ratio peaks obtained from the characterization of the gel extracted purified band. The peaks were obtained with an autoflex TOF/TOF.


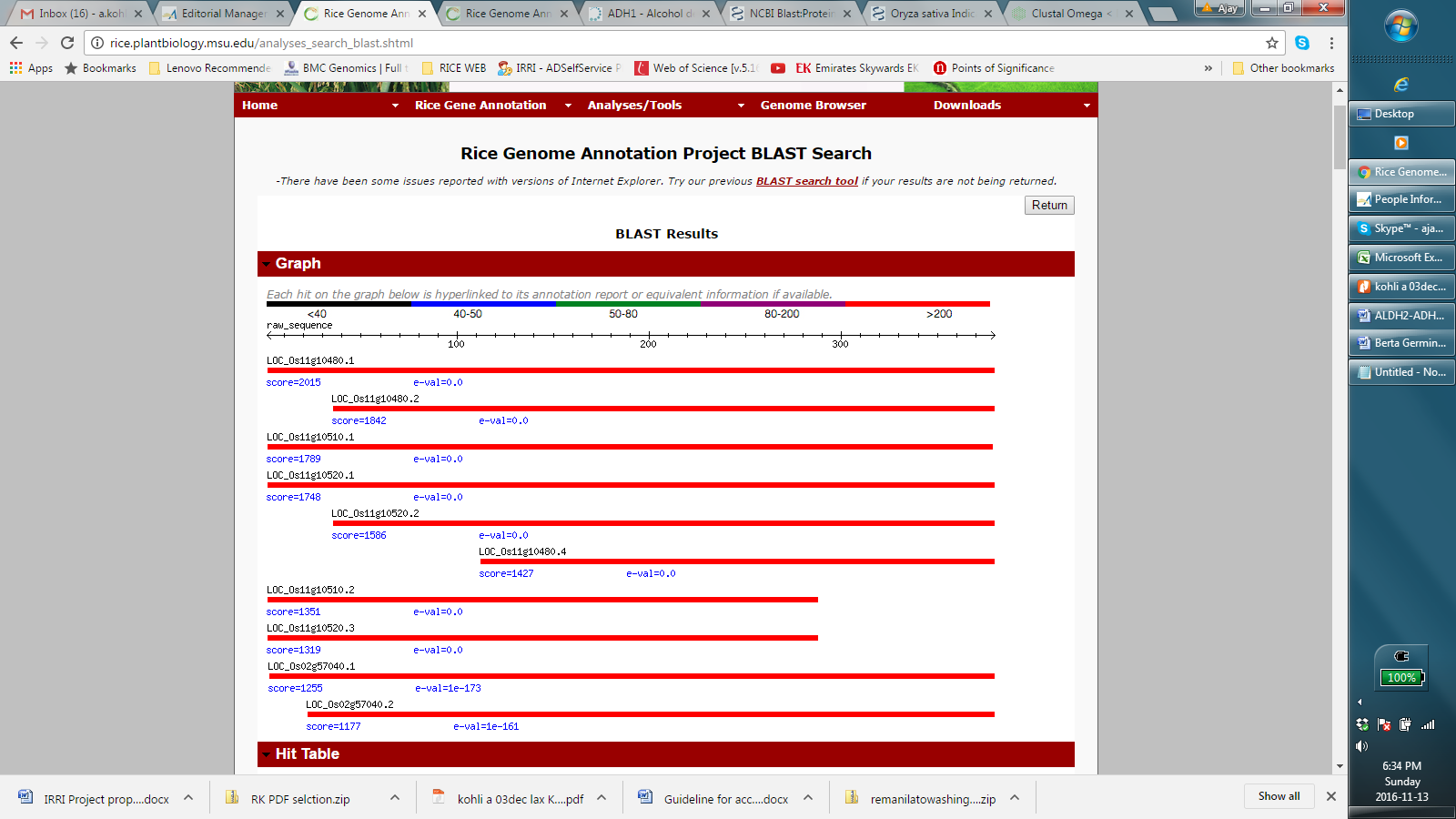


**Supplementary Figure 8. BLAST result using the MSU BLAST application.** The results of the BLAST show the peptides identified by MASCOT coincidence with different isoforms of ADH1 (*LOC_Os11g10480*) and an Alcohol dehydrogenase (fragment; *LOC_Os11g10520*).


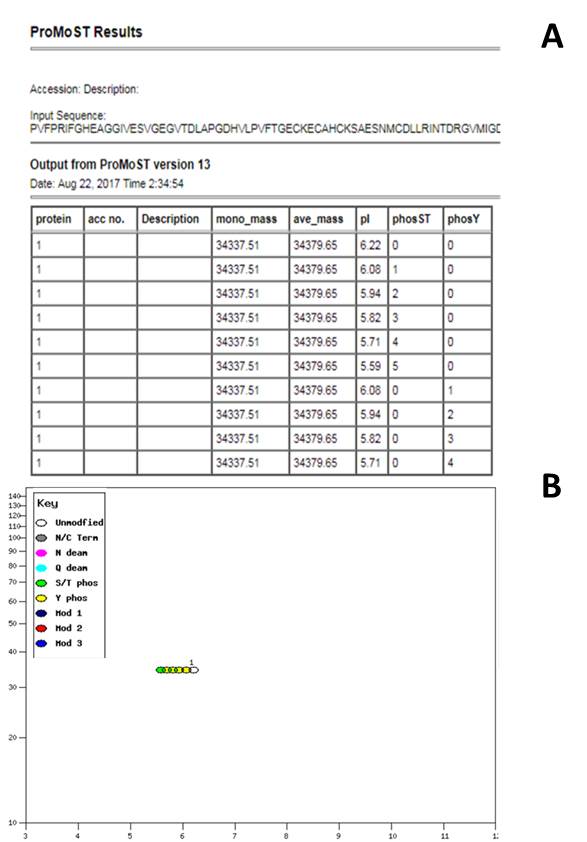


Supplementary Figure 9. **(A)** Online prediction and 2D gel image generated for the phosphorylated moieties of the rice ADH1 showing a series of spots for sequentially decreasing protein phosphorylation going from pH 5.59 to 6.0 around *Mw* of 34 kDa. **(B)** Predicted details of pH and *Mw* for the multi-phosphorylated rice ADH1.


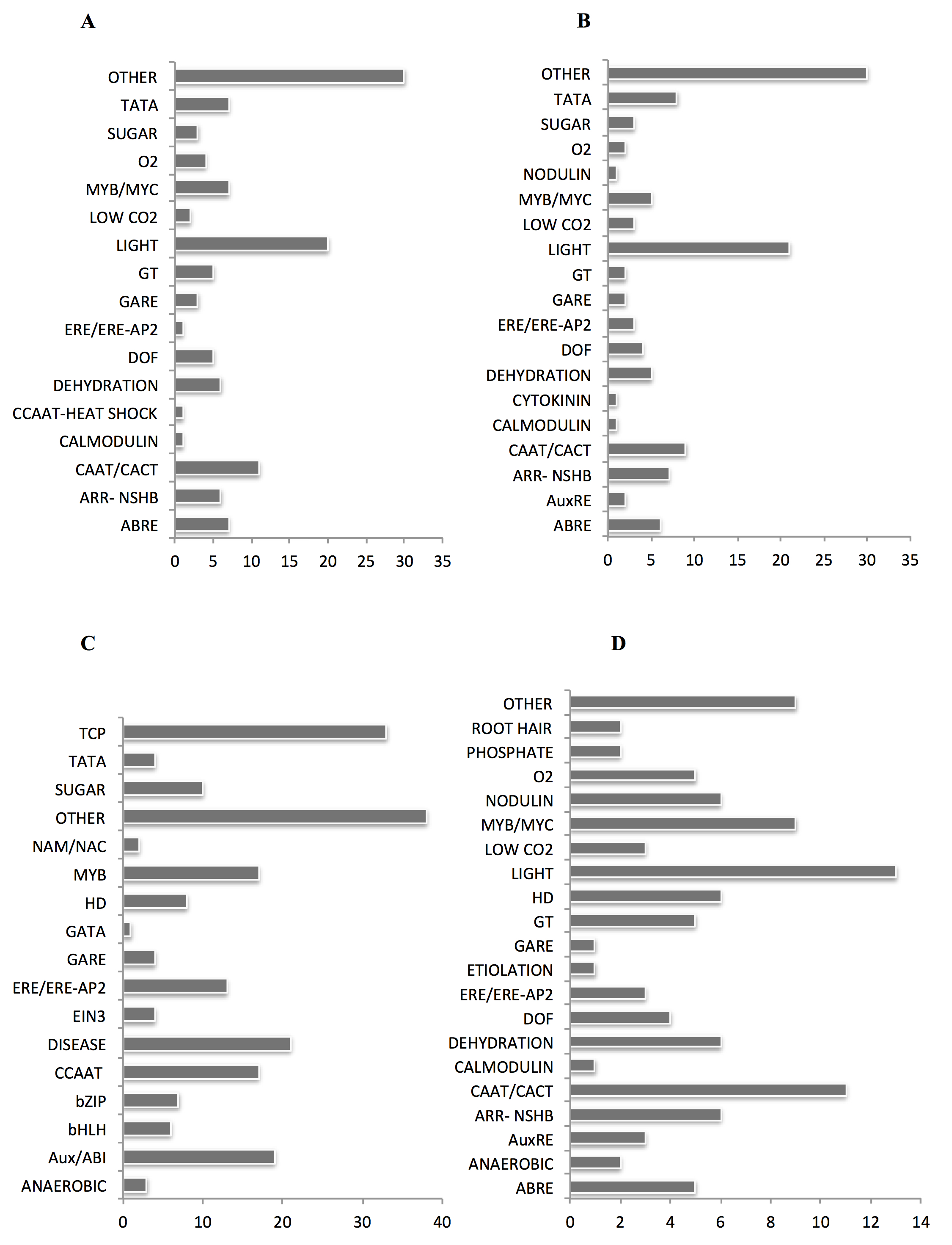


**Supplementary Figure 10.** Distribution of transcription factors in the promoter region 1kb upstream of ADH1 from ‘Nipponbare’/IR42 (A) and KHO (B) and ALDH2a from Nipponbare (C), and KHO (D).


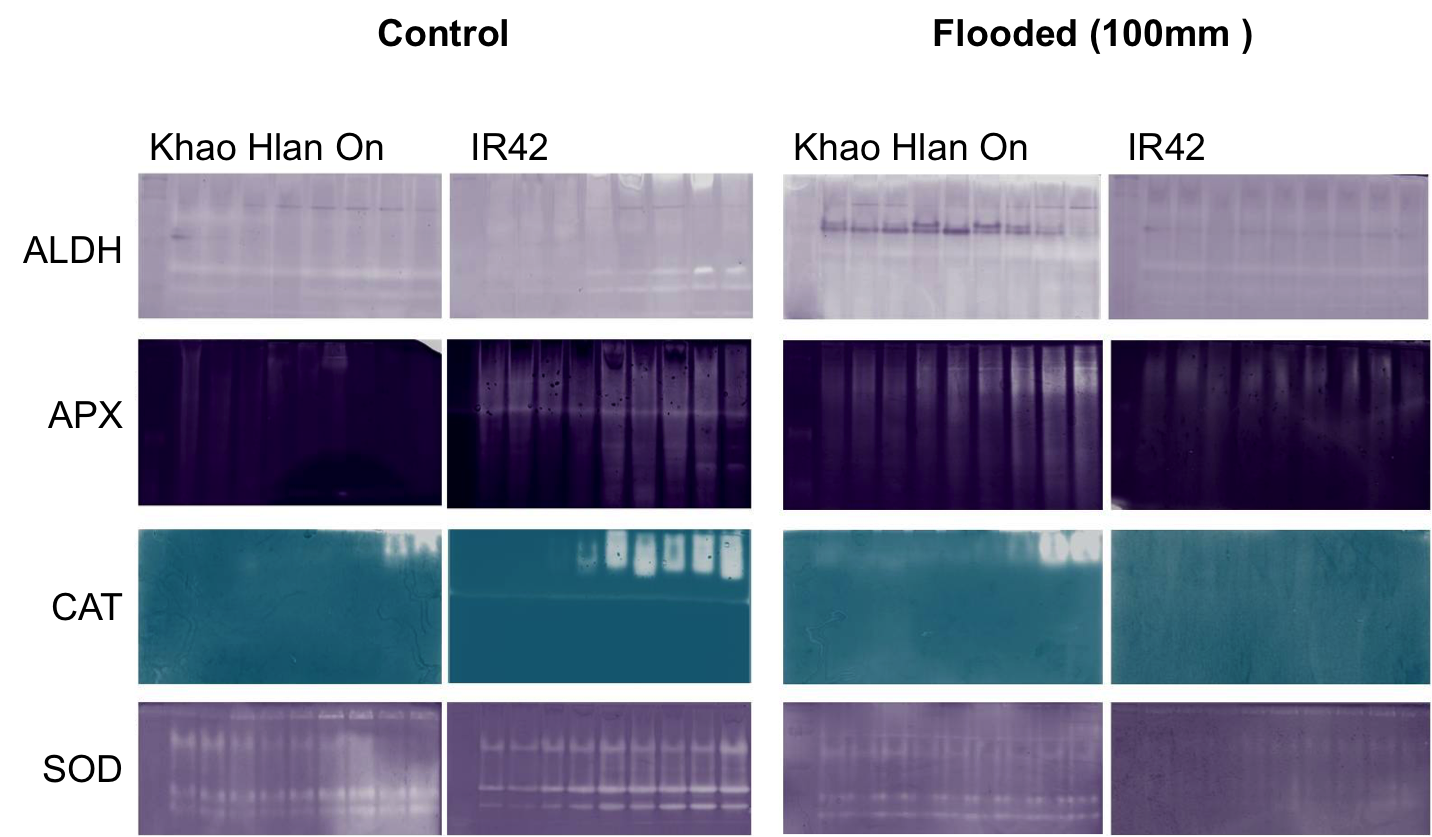


**Supplementary Figure 11. *In gel* assays for ALDH, APX, CAT and SOD enzymes in KHO and IR42 in 0 and 100 mm flooding.** Assays were carried out for 5 of the detoxifying and redox enzymes: aldehyde dehydrogenase (ALDH, E.C. 1.2.1.5.) involved in the acetaldehyde detoxification; ascorbate peroxidase (APX, E.C. 1.11.1.11)) involved in H_2_O_2_ detoxification; catalase (CAT, E.C. 1.11.1.6) involved in H_2_O_2_ detoxification also; and superoxide dismutase (SOD, E.C. 1.15.1.1) involved in radical O^-^_2_ detoxification.

Supplementary Tables

**Supplementary Table 1.** List of the genotypes analyzed in the physiology experiment.

| **Genotype** | **Subpopulation** |
| --- | --- |
| He Jiang 16 | *indica* |
| Kita Ake | *temperate japonica* |
| Nipponbare | *temperate japonica* |
| Moroberekan | *tropical japonica* |
| Azucena | *tropical japonica* |
| Apo | *aus* |
| Dular | *admix* |
| N22 | *aus* |
| Senia | *temperate japonica* |
| Bomba | *temperate japonica* |
| Amaroo | *temperate japonica* |
| Vandana | *indica* |
| Arborio | *japonica mix* |
| CO39 | *indica* |
| Khao Hlan On | *indica* |
| Mazhan (red) | *indica* |
| Khaiyan (Bangladesh) | *indica* |
| IR42 | *indica* |
| FR13a | *indica aus* |
| Daw Hawm | *indica aus* |
| IR64 | *indica* |

**Supplementary Table 2. Morpho-physiological features measured and measuring unit.**

| Parameter name | **Abbreviations** | **Description** | **Unit** |
| --- | --- | --- | --- |
| No. leaves above water | NLAW | Number of leaves emerging above water level per seedling | cm |
| No. plants surfacing aboce water | PAW |  |  |
| Number of plants | PN |  |  |
| Number of emerging plants | NEP |  |  |
| Leaf portion above water | LAW | Length of the leaf portion emerging above water level (submerged samples only) | cm |
| Total no. leaves | LN | Total number of leaves per seedling |  |
| Mesocotyl length | ML | Mesocotyl length from seed caryopsis | cm |
| Seedling base diameter | BD | Seedling base diameter measured above seed caryopsis | mm |
| Length first internode | IL | Length of first internode from mesocotyl to next internode | cm |
| Number of internodes | IN | Total number of internodes per seedling |  |
| Prophyllum length | PL | Prophyllum length | mm |
| Total shoot length | SL | Total shoot length (of longest leaf) | cm |
| Root length | RL | Root length (of longest root) | cm |
| Aerial parts dry weight | ADW | Aerial parts dry weight | mg |
| Root parts dry weight | RDW | Root parts dry weight | mg |
| Root length | LENGTH | Cumulative root length | cm |
| Root volume | ROOTVOL | Root volume | cm^3^ |
| Root projected area | PROJAREA | Projected area | cm^2^ |
| Root surface area | SURFAREA | Surface area | cm^2^ |
| Root average diameter | AVGDIAM | Average diameter | mm |
| Root length/ volume | LENVOL | Ratio of the cumulative root length divided by the volume analyzed | cm-^2^ |
| Root tips number | TIPS |  |  |
| Root forks number | FORKS |  |  |
| Root crosses number | CROSS |  |  |

| **Supplementary table 3A.** One-way ANOVA for the aerial traits analyzed, classified by trait | | | | | | |
| --- | --- | --- | --- | --- | --- | --- |
| Signif. codes: 0 ‘***’ 0.001 ‘**’ 0.01 ‘*’ 0.05 ‘.’ 0.1 ‘ ’ 1 | | | |  |  |  |
| **LN** |  |  |  |  |  |  |
|  | **Df** | **Sum Sq** | **Mean Sq** | **F value** | **Pr(>F)** |  |
| Variety | 20 | 34.055 | 1.70275 | 11.5441 | < 2.2e-16 | *** |
| Treatment | 1 | 0.047 | 0.04737 | 0.3211 | 0.5717 |  |
| Variety:Treatment | 18 | 8.853 | 0.49181 | 3.3343 | 2.26E-05 | *** |
| Residuals | 160 | 23.6 | 0.1475 |  |  |  |
|  |  |  |  |  |  |  |
| **PN** |  |  |  |  |  |  |
|  | **Df** | **Sum Sq** | **Mean Sq** | **F value** | **Pr(>F)** |  |
| Variety | 20 | 11126.9 | 556.34 | 210.338 | < 2.2e-16 | *** |
| Treatment | 1 | 2289.2 | 2289.16 | 865.467 | < 2.2e-16 | *** |
| Variety:Treatment | 18 | 2790.5 | 155.03 | 58.611 | < 2.2e-16 | *** |
| Residuals | 160 | 423.2 | 2.64 |  |  |  |
|  |  |  |  |  |  |  |
| **ML** |  |  |  |  |  |  |
|  | **Df** | **Sum Sq** | **Mean Sq** | **F value** | **Pr(>F)** |  |
| Variety | 20 | 201.178 | 10.059 | 32.803 | < 2.2e-16 | *** |
| Treatment | 1 | 204.989 | 204.989 | 668.489 | < 2.2e-16 | *** |
| Variety:Treatment | 18 | 95.636 | 5.313 | 17.326 | < 2.2e-16 | *** |
| Residuals | 160 | 49.063 | 0.307 |  |  |  |
|  |  |  |  |  |  |  |
| **BD** |  |  |  |  |  |  |
|  | **Df** | **Sum Sq** | **Mean Sq** | **F value** | **Pr(>F)** |  |
| Variety | 20 | 5.6877 | 0.284387 | 5.2981 | 4.25E-10 | *** |
| Treatment | 1 | 0.0304 | 0.030442 | 0.5671 | 0.452505 |  |
| Variety:Treatment | 18 | 2.4481 | 0.136004 | 2.5338 | 0.001081 | ** |
| Residuals | 160 | 8.5883 | 0.053677 |  |  |  |
|  |  |  |  |  |  |  |
| **IL** |  |  |  |  |  |  |
|  | **Df** | **Sum Sq** | **Mean Sq** | **F value** | **Pr(>F)** |  |
| Variety | 20 | 477.44 | 23.87 | 6.2438 | 4.00E-12 | *** |
| Treatment | 1 | 728.2 | 728.2 | 190.4601 | < 2.2e-16 | *** |
| Variety:Treatment | 18 | 224.8 | 12.49 | 3.2665 | 3.15E-05 | *** |
| Residuals | 160 | 611.74 | 3.82 |  |  |  |
|  |  |  |  |  |  |  |
| **IN** |  |  |  |  |  |  |
|  | **Df** | **Sum Sq** | **Mean Sq** | **F value** | **Pr(>F)** |  |
| Variety | 20 | 14.6 | 0.73 | 0.7053 | 0.8168 |  |
| Treatment | 1 | 0.132 | 0.13158 | 0.1271 | 0.7219 |  |
| Variety:Treatment | 18 | 13.168 | 0.73158 | 0.7068 | 0.8007 |  |
| Residuals | 160 | 165.6 | 1.035 |  |  |  |
| **PL** |  |  |  |  |  |  |
|  | **Df** | **Sum Sq** | **Mean Sq** | **F value** | **Pr(>F)** |  |
| Variety | 20 | 2528 | 126.4 | 17.955 | < 2.2e-16 | *** |
| Treatment | 1 | 1316.1 | 1316.1 | 186.948 | < 2.2e-16 | *** |
| Variety:Treatment | 18 | 2445.8 | 135.88 | 19.301 | < 2.2e-16 | *** |
| Residuals | 160 | 1126.4 | 7.04 |  |  |  |
| **SL** |  |  |  |  |  |  |
|  | **Df** | **Sum Sq** | **Mean Sq** | **F value** | **Pr(>F)** |  |
| Variety | 20 | 2596.4 | 129.82 | 7.4277 | 1.53E-14 | *** |
| Treatment | 1 | 2238.42 | 2238.42 | 128.0713 | < 2.2e-16 | *** |
| Variety:Treatment | 18 | 942.69 | 52.37 | 2.9965 | 0.0001174 | *** |
| Residuals | 160 | 2796.47 | 17.48 |  |  |  |
|  |  |  |  |  |  |  |
| **ADW** |  |  |  |  |  |  |
|  | **Df** | **Sum Sq** | **Mean Sq** | **F value** | **Pr(>F)** |  |
| Variety | 20 | 4021.9 | 201.1 | 4.9471 | 2.51E-09 | *** |
| Treatment | 1 | 830.9 | 830.88 | 20.4401 | 1.19E-05 | *** |
| Variety:Treatment | 18 | 2021 | 112.28 | 2.7621 | 0.0003641 | *** |
| Residuals | 160 | 6503.9 | 40.65 |  |  |  |
|  |  |  |  |  |  |  |
| **RDW** |  |  |  |  |  |  |
|  | **Df** | **Sum Sq** | **Mean Sq** | **F value** | **Pr(>F)** |  |
| Variety | 20 | 377.73 | 18.886 | 5.1647 | 8.32E-10 | *** |
| Treatment | 1 | 189.15 | 189.151 | 51.7253 | 2.32E-11 | *** |
| Variety:Treatment | 18 | 111.07 | 6.171 | 1.6875 | 0.04637 | * |
| Residuals | 160 | 585.09 | 3.657 |  |  |  |

**Supplementary table 3B**. One-way ANOVA for the root traits analyzed, classified by trait

| (Signif. codes: 0 ‘***’ 0.001 ‘**’ 0.01 ‘*’ 0.05 ‘.’ 0.1 ‘ ’ 1) |  |  |  |
| --- | --- | --- | --- |

| **Response LENGTH :** |  |  |  |  |  |  |
| --- | --- | --- | --- | --- | --- | --- |
|  | **Df** | **Sum Sq** | **Mean Sq** | **F value** | **Pr(>F)** |  |
| TRTMENT | 1 | 471020 | 471020 | 114.4 | < 2.2e-16 | *** |
| GTYPE | 20 | 1139429 | 56971 | 13.8371 | < 2.2e-16 | *** |
| TRTMENT:GTYPE | 20 | 174972 | 8749 | 2.1248 | 0.006411 | ** |
| Residuals | 126 | 518781 | 4117 |  |  |  |
| --- |  |  |  |  |  |  |
| **Response PROJAREA :** | |  |  |  |  |  |
|  | **Df** | **Sum Sq** | **Mean Sq** | **F value** | **Pr(>F)** |  |
| TRTMENT | 1 | 99.794 | 99.794 | 121.8797 | < 2.2e-16 | *** |
| GTYPE | 20 | 268.005 | 13.4 | 16.3659 | < 2.2e-16 | *** |
| TRTMENT:GTYPE | 20 | 37.248 | 1.862 | 2.2746 | 0.003207 | ** |
| Residuals | 126 | 103.167 | 0.819 |  |  |  |
| --- |  |  |  |  |  |  |
|  | | | |  |  |  |
| **Response SURFAREA :** | |  |  |  |  |  |
|  | **Df** | **Sum Sq** | **Mean Sq** | **F value** | **Pr(>F)** |  |
| TRTMENT | 1 | 984.93 | 984.93 | 121.88 | < 2.2e-16 | *** |
| GTYPE | 20 | 2645.11 | 132.26 | 16.366 | < 2.2e-16 | *** |
| TRTMENT:GTYPE | 20 | 367.62 | 18.38 | 2.2745 | 0.003207 | ** |
| Residuals | 126 | 1018.22 | 8.08 |  |  |  |
| --- |  |  |  |  |  |  |
|  |  |  |  |  |  |  |
| **Response AVGDIAM** | **:** |  |  |  |  |  |
|  | **Df** | **Sum Sq** | **Mean Sq** | **F value** | **Pr(>F)** |  |
| TRTMENT | 1 | 0.000026 | 0.0000255 | 0.0958 | 0.7574 |  |
| GTYPE | 20 | 0.0613 | 0.003065 | 11.5025 | <2e-16 | *** |
| TRTMENT:GTYPE | 20 | 0.088277 | 0.0044138 | 16.5645 | <2e-16 | *** |
| Residuals | 126 | 0.033574 | 0.0002665 |  |  |  |
| **Response LENVOL :** |  |  |  |  |  |  |
|  | **Df** | **Sum Sq** | **Mean Sq** | **F value** | **Pr(>F)** |  |
| TRTMENT | 1 | 5.23E+10 | 5.23E+10 | 114.4 | < 2.2e-16 | *** |
| GTYPE | 20 | 1.27E+11 | 6.33E+09 | 13.8371 | < 2.2e-16 | *** |
| TRTMENT:GTYPE | 20 | 1.94E+10 | 9.72E+08 | 2.1248 | 0.006411 | ** |
| Residuals | 126 | 5.76E+10 | 4.57E+08 |  |  |  |
| **Response ROOTVOL :** | |  |  |  |  |  |
|  | **Df** | **Sum Sq** | **Mean Sq** | **F value** | **Pr(>F)** |  |
| TRTMENT | 1 | 7426.7 | 7426.7 | 63.529 | 8.16E-13 | *** |
| GTYPE | 20 | 30955.5 | 1547.8 | 13.2398 | < 2.2e-16 | *** |
| TRTMENT:GTYPE | 20 | 5424.9 | 271.2 | 2.3203 | 0.002589 | ** |
| Residuals | 126 | 14729.7 | 116.9 |  |  |  |
| **Response TIPS :** |  |  |  |  |  |  |
|  | **Df** | **Sum Sq** | **Mean Sq** | **F value** | **Pr(>F)** |  |
| TRTMENT | 1 | 50163 | 50163 | 45.3606 | 5.22E-10 | *** |
| GTYPE | 20 | 131263 | 6563 | 5.9348 | 1.03E-10 | *** |
| TRTMENT:GTYPE | 20 | 46294 | 2315 | 2.0931 | 0.007411 | ** |
| Residuals | 126 | 139340 | 1106 |  |  |  |
| **Response FORKS :** |  |  |  |  |  |  |
|  | **Df** | **Sum Sq** | **Mean Sq** | **F value** | **Pr(>F)** |  |
| TRTMENT | 1 | 266794926 | 266794926 | 209.9649 | <2e-16 | *** |
| GTYPE | 20 | 273700094 | 13685005 | 10.77 | <2e-16 | *** |
| TRTMENT:GTYPE | 20 | 29489932 | 1474497 | 1.1604 | 0.2997 |  |
| Residuals | 126 | 160103748 | 1270665 |  |  |  |
|  |  |  |  |  |  |  |
| **Response CROSS :** |  |  |  |  |  |  |
|  | **Df** | **Sum Sq** | **Mean Sq** | **F value** | **Pr(>F)** |  |
| TRTMENT | 1 | 28913883 | 28913883 | 165.0084 | < 2.2e-16 | *** |
| GTYPE | 20 | 32092801 | 1604640 | 9.1575 | 2.34E-16 | *** |
| TRTMENT:GTYPE | 20 | 3949122 | 197456 | 1.1269 | 0.3311 |  |
| Residuals | 126 | 22078569 | 175227 |  |  |  |
|  |  |  |  |  |  |  |

| **Supplementary Table 4A.** Table with the means for all the aerial traits analyzed for each variety, and Kruskal Wallis parameters test: chi-squared, degrees of freedom (df) and p-values | | | | | | | | | | | | |  |  |
| --- | --- | --- | --- | --- | --- | --- | --- | --- | --- | --- | --- | --- | --- | --- |
|  | **ADW** | **BD** | **FP** | **IL** | **IN** | **LAW** | **LN** | **ML** | **NEP** | **PAW** | **PL** | **PN** | **RDW** | **SL** |
| **Amaroo** | 31.44 | 1.81 | 5.00 | 14.78 | 1.00 | 1.80 | 2.60 | 2.84 | 22.00 | 23.80 | 19.16 | 22.00 | 6.98 | 33.32 |
| **Apo** | 29.10 | 1.29 | 1.00 | 13.26 | 1.00 | 2.20 | 3.00 | 0.00 | 28.00 | 22.50 | 5.89 | 28.00 | 4.60 | 32.68 |
| **Arborio** | 26.32 | 1.51 | 3.00 | 13.74 | 1.00 | 2.00 | 2.80 | 4.04 | 20.00 | 30.10 | 13.13 | 25.00 | 7.20 | 31.70 |
| **Azucena** | 28.00 | 1.54 | 2.00 | 13.28 | 1.00 | 2.00 | 2.00 | 4.24 | 28.00 | 26.80 | 5.93 | 30.00 | 5.64 | 35.80 |
| **Bomba** | 31.08 | 1.73 | 1.00 | 15.80 | 1.00 | 2.00 | 3.00 | 3.48 | 27.00 | 27.40 | 23.95 | 29.00 | 8.26 | 37.00 |
| **CO39** | 36.42 | 1.80 | 3.00 | 19.20 | 1.00 | 2.40 | 3.00 | 2.86 | 15.00 | 20.20 | 27.51 | 20.00 | 5.64 | 41.66 |
| **Daw Hawm** | 16.02 | 1.19 | 2.00 | 12.30 | 1.00 | 1.20 | 2.20 | 2.78 | 23.00 | 14.40 | 9.94 | 2.70 | 3.28 | 26.20 |
| **Dular** | 17.76 | 1.19 | 0.00 | 13.65 | 0.80 | 1.80 | 2.00 | 3.02 | 18.00 | 15.60 | 8.93 | 21.00 | 3.42 | 30.42 |
| **FR13A** | 33.50 | 1.68 | 2.00 | 14.48 | 1.00 | 3.00 | 3.00 | 3.15 | 14.00 | 26.10 | 10.95 | 17.00 | 5.74 | 35.40 |
| **He Jiang16** | 23.56 | 1.66 | 2.00 | 13.23 | 0.80 | 2.40 | 3.80 | 2.52 | 10.00 | 11.90 | 19.41 | 11.00 | 5.74 | 27.26 |
| **IR42** | 11.84 | 1.19 | 4.00 | 9.64 | 1.00 | 1.60 | 2.20 | 2.78 | 4.00 | 9.38 | 11.54 | 8.00 | 2.75 | 22.10 |
| **Khaiyan** | 33.12 | 1.53 | 11.00 | 16.18 | 0.80 | 2.80 | 2.80 | 2.90 | 23.00 | 28.00 | 10.95 | 34.00 | 5.64 | 38.80 |
| **Khao Hlan On** | 30.84 | 1.55 | 11.00 | 17.56 | 1.00 | 2.80 | 2.80 | 2.60 | 20.00 | 25.40 | 25.91 | 33.00 | 5.90 | 38.74 |
| **Kita Ake** | 32.22 | 1.61 | 3.00 | 15.66 | 1.00 | 2.60 | 3.60 | 2.40 | 12.00 | 20.30 | 18.66 | 13.00 | 7.02 | 31.70 |
| **Ma Zhan (red)** | 23.66 | 1.43 | 11.00 | 13.54 | 1.00 | 2.00 | 2.00 | 2.52 | 20.60 | 24.60 | 12.20 | 32.00 | 4.76 | 32.72 |
| **Moroberekan** | 24.72 | 1.56 | 0.00 | 15.88 | 1.00 | 2.00 | 2.00 | 3.52 | 22.00 | 28.70 | 8.79 | 23.00 | 5.90 | 34.42 |
| **N22** | 19.98 | 1.24 | 1.00 | 12.48 | 1.00 | 1.40 | 2.40 | 0.00 | 31.00 | 21.00 | 24.32 | 33.00 | 5.50 | 29.44 |
| **Nipponbare** | 22.58 | 1.66 | 7.00 | 12.74 | 1.00 | 1.80 | 2.80 | 2.47 | 17.00 | 21.80 | 22.43 | 17.00 | 3.92 | 29.32 |
| **Senia** | 34.04 | 1.95 | 0.00 | 16.06 | 1.00 | 2.00 | 3.00 | 3.89 | 17.00 | 28.70 | 22.09 | 19.00 | 8.16 | 36.30 |
| **Vandana** | 27.98 | 1.63 | 5.00 | 13.46 | 1.00 | 2.20 | 3.00 | 0.00 | 21.00 | 21.50 | 13.13 | 23.00 | 6.58 | 33.14 |
| **chi-squared** | 48.15 | 48.55 | 99.00 | 61.38 | 17.35 | 52.90 | 72.04 | 60.63 | 98.79 | 71.32 | 85.61 | 99.00 | 54.43 | 60.50 |
| **Df** | 19 | 19 | 19 | 19 | 19 | 19 | 19 | 19 | 19 | 19 | 19 | 19 | 19 | 19 |
| **p-value** | 0.000244 | 0.0002138 | 8.12E-12 | 2.33E-06 | 0.5661 | 4.85E-05 | 4.20E-08 | 3.08E-06 | 8.88E-13 | 5.55E-08 | 1.97E-10 | 8.12E-12 | 2.84E-05 | 3.22E-06 |

**Supplementary table 4B.** Table with the means for all the root traits analyzed for each variety, and Kruskal Wallis parameters test: chi-squared, degrees of freedom (df) and p-values

|  | **AVGDIAM** | **CROSS** | **FORKS** | **LENGTH** | **LENVOL** | **PROJAREA** | **ROOTVOL** | **SURFAREA** | **TIPS** |
| --- | --- | --- | --- | --- | --- | --- | --- | --- | --- |
| **Amaroo** | 0.1931 | 564.25 | 2411.25 | 234.7698 | 78256.6 | 4.5567 | 0.0695 | 14.3153 | 2837.25 |
| **Apo** | 0.1641 | 557.5 | 2052.5 | 216.3341 | 72111.37 | 3.47755 | 0.04425 | 10.925025 | 2690.25 |
| **Arborio** | 0.166525 | 974.25 | 3457.5 | 347.8226 | 115940.88 | 5.540575 | 0.07175 | 17.40625 | 4575 |
| **Azucena** | 0.1663 | 692 | 2573.75 | 253.233 | 84411 | 4.22235 | 0.05525 | 13.264875 | 2717.5 |
| **Bomba** | 0.154225 | 1292.25 | 4366.5 | 382.6219 | 127540.63 | 5.85995 | 0.071 | 18.40955 | 4490.5 |
| **CO39** | 0.1667 | 606.75 | 2307 | 215.4474 | 71815.81 | 3.504725 | 0.0455 | 11.010425 | 2598 |
| **Daw Hawm** | 0.165125 | 731 | 2761.25 | 293.6085 | 97869.49 | 4.835175 | 0.0625 | 15.1901 | 3214.25 |
| **Dular** | 0.18485 | 296 | 1158.5 | 119.5169 | 39838.97 | 2.1015 | 0.0295 | 6.60205 | 1232.75 |
| **FR13a** | 0 | 0 | 0 | 0 | 0 | 0 | 0 | 0 | 0 |
| **He Jiang 16** | 0.15825 | 981.75 | 3569.25 | 319.0209 | 106340.31 | 5.03575 | 0.06275 | 15.82025 | 4089.75 |
| **IR42** | 0.143175 | 259.5 | 975.25 | 107.3198 | 35773.26 | 1.55305 | 0.0185 | 4.879 | 1497.5 |
| **IR64** | 0.1554 | 367.5 | 1432.5 | 160.484 | 53494.67 | 2.484775 | 0.03075 | 7.806125 | 2204 |
| **Khaiyan** | 0.198625 | 548.5 | 2357.25 | 239.1219 | 79707.32 | 4.5681 | 0.0705 | 14.351125 | 2944.5 |
| **Khao Hlan On** | 0.131075 | 2236.25 | 6757.75 | 512.5349 | 170844.96 | 6.65325 | 0.0685 | 20.901775 | 6819 |
| **Kita Ake** | 0.177675 | 669.25 | 2611.75 | 249.9705 | 83323.51 | 4.423125 | 0.0615 | 13.89565 | 2729.5 |
| **Ma Zhan (red)** | 0.158875 | 640 | 2263.75 | 231.1853 | 77061.75 | 3.6801 | 0.04625 | 11.561375 | 2634.75 |
| **Moroberekan** | 0.1694 | 954.75 | 3335.75 | 289.5598 | 96519.94 | 4.86525 | 0.06425 | 15.284725 | 3558.5 |
| **N22** | 0.152925 | 614 | 2329.5 | 231.7067 | 77235.57 | 3.48395 | 0.04175 | 10.945175 | 3663.25 |
| **Nipponbare** | 0.153225 | 737.25 | 2570.25 | 273.4491 | 91149.69 | 4.121725 | 0.04925 | 12.94885 | 2837.5 |
| **Senia** | 0.1885 | 680 | 3027.5 | 310.9551 | 103651.7 | 5.88065 | 0.0875 | 18.474625 | 3393.5 |
| **Vandana** | 0.169875 | 591 | 2383.75 | 232.1389 | 77379.63 | 3.917075 | 0.05225 | 12.305825 | 2887.25 |
| **chi-squared** | 45.535 | 51.963 | 53.992 | 59.468 | 59.468 | 65.411 | 63.363 | 65.411 | 55.636 |
| **Df** | 20 | 20 | 20 | 20 | 20 | 20 | 20 | 20 | 20 |
| **p-value** | 0.0009332 | 0.0001153 | 5.80E-05 | 8.61E-06 | 8.61E-06 | 1.00E-06 | 2.12E-06 | 1.00E-06 | 3.30E-05 |

**Supplementary Table 5.** Protein identification mascot results and protein modification predictions.

| **1.** | ADH1_ORYSI    Mass: 40958    Score: 71     Expect: 0.00035  Matches: 10 |
| --- | --- |
|  | Alcohol dehydrogenase 1 OS=Oryza sativa subsp. indica GN=ADH1 PE=2 SV=1 |
|  | ADH1_ORYSJ    Mass: 40958    Score: 71     Expect: 0.00035  Matches: 10 |
|  | Alcohol dehydrogenase 1 OS=Oryza sativa subsp. japonica GN=ADH1 PE=2 SV=2 |
| **2.** | CADH6_ORYSJ    Mass: 39061    Score: 36     Expect: 1.1  Matches: 6 |
|  | Probable cinnamyl alcohol dehydrogenase 6 OS=Oryza sativa subsp. japonica GN=CAD6 PE=2 SV=2 |
| **3.** | BRK1_ORYSJ    Mass: 9570     Score: 20     Expect: 45  Matches: 2 |
|  | Probable protein BRICK1 OS=Oryza sativa subsp. japonica GN=Os02g0829900 PE=3 SV=1 |

**Supplementary Table 6.** Prediction of O-Glycosylation sites in Khao Hlan On and Nipponbare. Software output that predicts O-glycosylation sites from a sequence, a threshold above 0.5 is considered potentially O-glycosylated.

| **Nipponbare** | | |  | **Khao Hlan On** | | | | **Khao Hlan On truncated sequence** | | | |
| --- | --- | --- | --- | --- | --- | --- | --- | --- | --- | --- | --- |
| start | end | score | comment | start | end | score | comment | start | end | score | comment |
| 3 | 3 | 0.21 |  | 3 | 3 | 0.31 |  | 3 | 3 | 0.37 |  |
| 44 | 44 | 0.00 |  | 37 | 37 | 0.01 |  | 37 | 37 | 0.01 |  |
| 45 | 45 | 0.03 |  | 46 | 46 | 0.00 |  | 46 | 46 | 0.00 |  |
| 49 | 49 | 0.02 |  | 47 | 47 | 0.05 |  | 47 | 47 | 0.05 |  |
| 60 | 60 | 0.13 |  | 51 | 51 | 0.02 |  | 51 | 51 | 0.02 |  |
| 77 | 77 | 0.02 |  | 62 | 62 | 0.13 |  | 62 | 62 | 0.14 |  |
| 83 | 83 | 0.14 |  | 79 | 79 | 0.02 |  | 79 | 79 | 0.03 |  |
| 96 | 96 | 0.11 |  | 85 | 85 | 0.15 |  | 85 | 85 | 0.16 |  |
| 107 | 107 | 0.08 |  | 98 | 98 | 0.11 |  | 98 | 98 | 0.12 |  |
| 110 | 110 | 0.34 |  | 109 | 109 | 0.11 |  | 109 | 109 | 0.14 |  |
| 120 | 120 | 0.55 | #POSITIVE | 112 | 112 | 0.26 |  | 112 | 112 | 0.29 |  |
| 131 | 131 | 0.15 |  | 116 | 116 | 0.22 |  | 116 | 116 | 0.23 |  |
| 134 | 134 | 0.20 |  | 124 | 124 | 0.59 | #POSITIVE | 124 | 124 | 0.62 | #POSITIVE |
| 146 | 146 | 0.01 |  | 131 | 131 | 0.52 | #POSITIVE | 131 | 131 | 0.50 | #POSITIVE |
| 147 | 147 | 0.09 |  | 137 | 137 | 0.23 |  | 137 | 137 | 0.24 |  |
| 148 | 148 | 0.02 |  | 140 | 140 | 0.24 |  | 140 | 140 | 0.24 |  |
| 150 | 150 | 0.05 |  | 152 | 152 | 0.02 |  | 152 | 152 | 0.02 |  |
| 153 | 153 | 0.02 |  | 153 | 153 | 0.09 |  | 153 | 153 | 0.13 |  |
| 176 | 176 | 0.02 |  | 154 | 154 | 0.02 |  | 154 | 154 | 0.02 |  |
| 180 | 180 | 0.20 |  | 156 | 156 | 0.05 |  | 156 | 156 | 0.06 |  |
| 181 | 181 | 0.03 |  | 159 | 159 | 0.06 |  | 159 | 159 | 0.07 |  |
| 186 | 186 | 0.14 |  | 163 | 163 | 0.03 |  | 163 | 163 | 0.04 |  |
| 196 | 196 | 0.04 |  | 184 | 184 | 0.02 |  | 184 | 184 | 0.02 |  |
| 197 | 197 | 0.07 |  | 188 | 188 | 0.20 |  | 188 | 188 | 0.19 |  |
| 220 | 220 | 0.03 |  | 189 | 189 | 0.03 |  | 189 | 189 | 0.03 |  |
| 241 | 241 | 0.07 |  | 194 | 194 | 0.13 |  | 194 | 194 | 0.14 |  |
| 261 | 261 | 0.13 |  | 204 | 204 | 0.05 |  | 204 | 204 | 0.05 |  |
| 268 | 268 | 0.02 |  | 205 | 205 | 0.07 |  | 205 | 205 | 0.07 |  |
| 272 | 272 | 0.01 |  | 228 | 228 | 0.03 |  | 228 | 228 | 0.03 |  |
| 306 | 306 | 0.06 |  | 246 | 246 | 0.03 |  | 246 | 246 | 0.02 |  |
| 316 | 316 | 0.09 |  | 251 | 251 | 0.05 |  | 251 | 251 | 0.05 |  |
| 320 | 320 | 0.06 |  | 272 | 272 | 0.30 |  |  |  |  |  |
| 329 | 329 | 0.06 |  | 273 | 273 | 0.09 |  |  |  |  |  |
| 350 | 350 | 0.30 |  | 280 | 280 | 0.03 |  |  |  |  |  |
| 352 | 352 | 0.09 |  | 284 | 284 | 0.01 |  |  |  |  |  |
| 356 | 356 | 0.14 |  | 292 | 292 | 0.00 |  |  |  |  |  |
| 360 | 360 | 0.07 |  | 320 | 320 | 0.10 |  |  |  |  |  |
|  |  |  |  | 325 | 325 | 0.14 |  |  |  |  |  |
|  |  |  |  | 332 | 332 | 0.06 |  |  |  |  |  |
|  |  |  |  | 336 | 336 | 0.03 |  |  |  |  |  |
|  |  |  |  | 346 | 346 | 0.08 |  |  |  |  |  |
|  |  |  |  | 361 | 361 | 0.06 |  |  |  |  |  |
|  |  |  |  | 363 | 363 | 0.07 |  |  |  |  |  |
